# OHMM: a Hidden Markov Model accurately predicting the occupancy of a transcription factor with a self-overlapping binding motif

**DOI:** 10.1186/1471-2105-10-208

**Published:** 2009-07-07

**Authors:** Amar Drawid, Nupur Gupta, Vijayalakshmi H Nagaraj, Céline Gélinas, Anirvan M Sengupta

**Affiliations:** 1 BioMAPS Institute for Quantitative Biology, Rutgers University, Piscataway, NJ, USA; 2 Bioinformatics Group, sanofi-aventis U.S., Bridgewater, NJ, USA; 3 Center for Advanced Biotechnology and Medicine, Robert Wood Johnson Medical School, Piscataway, NJ, USA; 4 Graduate Program in Biochemistry and Molecular Biology, Robert Wood Johnson Medical School, Piscataway, NJ, USA; 5 Department of Biochemistry, Robert Wood Johnson Medical School, Piscataway, NJ, USA; 6 Department of Physics and Astronomy, Rutgers University, Piscataway, NJ, USA

## Abstract

**Background:**

DNA sequence binding motifs for several important transcription factors happen to be self-overlapping. Many of the current regulatory site identification methods do not explicitly take into account the overlapping sites. Moreover, most methods use arbitrary thresholds and fail to provide a biophysical interpretation of statistical quantities. In addition, commonly used approaches do not include the location of a site with respect to the transcription start site (TSS) in an integrated probabilistic framework while identifying sites. Ignoring these features can lead to inaccurate predictions as well as incorrect design and interpretation of experimental results.

**Results:**

We have developed a tool based on a Hidden Markov Model (HMM) that identifies binding location of transcription factors with preference for self-overlapping DNA motifs by combining the effects of their alternative binding modes. Interpreting HMM parameters as biophysical quantities, this method uses the occupancy probability of a transcription factor on a DNA sequence as the discriminant function, earning the algorithm the name OHMM: **O**ccupancy via **H**idden **M**arkov **M**odel. OHMM learns the classification threshold by training emission probabilities using unaligned sequences containing known sites and estimating transition probabilities to reflect site density in all promoters in a genome. While identifying sites, it adjusts parameters to model site density changing with the distance from the transcription start site. Moreover, it provides guidance for designing padding sequences in gel shift experiments. In the context of binding sites to transcription factor NF-κB, we find that the occupancy probability predicted by OHMM correlates well with the binding affinity in gel shift experiments. High evolutionary conservation scores and enrichment in experimentally verified regulated genes suggest that NF-κB binding sites predicted by our method are likely to be functional.

**Conclusion:**

Our method deals specifically with identifying locations with multiple overlapping binding sites by computing the local occupancy of the transcription factor. Moreover, considering OHMM as a biophysical model allows us to learn the classification threshold in a principled manner. Another feature of OHMM is that we allow transition probabilities to change with location relative to the TSS. OHMM could be used to predict physical occupancy, and provides guidance for proper design of gel-shift experiments. Based upon our predictions, new insights into NF-κB function and regulation and possible new biological roles of NF-κB were uncovered.

## Background

Identification of short, degenerate DNA sequences (sites) that bind to a transcription factor is a difficult problem [1]. The particular sub-problems of identification of sites corresponding to self-overlapping motifs, determination of a threshold for this purpose, biophysical interpretation of statistical quantities used in probabilistic site identification methods and estimation of occupancy of these sites by a transcription factor have not been addressed in detail before. These problems form the focus of this study.

Several transcription factors bind to self-overlapping DNA motifs. When a motif overlaps with itself with a particular shift, the transcription factor can bind in more than one sequence windows, i.e. to overlapping sites. Examples include *Drosophila *developmental transcription factor Hunchback [2], worm PHA-4 [3], human Sp-1, C/EBPalpha, yeast ADR1, MIG1, chicken Cdx-1, *Arabidopsis *Agamous, etc. [4]. Furthermore, when binding by the transcription factor in either orientation is permissible, the corresponding DNA site and its reverse complement can be considered as two different types of sites that are overlapping, as long as the motif is not exactly palindromic. Problems similar to the identification of overlapping sites also arise in the context of prediction of nucleosome positioning [5].

### Self-overlapping motif of NF-κB family of factors

**Figure 1:**
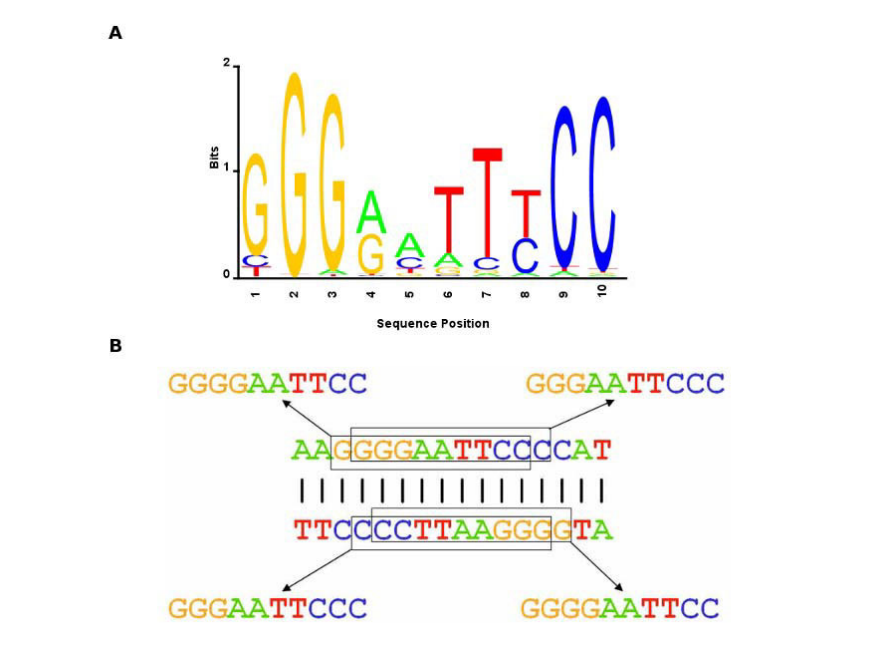
**Overlapping κB sites**. A. Sequence logo of the κB site, based upon the initial motif profile used in HMM training, where the overall height of the nucleotide stack at each position is proportional to the information content at that position and the height of each nucleotide within the stack is proportional to its frequency. B. Four overlapping κB sites are present on the two strands in three adjacent 10-base pair sequence windows.

The NF-κB family of transcription factors is a prominent example of transcription factors with self-overlapping motifs. These factors are key mediators of the cellular response to infection, injury, inflammation, or other stress conditions that lead to rapid alterations in cellular gene expression [6,7]. The vertebrate members include the c-Rel, RelA, RelB, NFKB1/p105 and NFKB2/p100 proteins that form homo- or heterodimers with one another and bind to similar sites. These NF-κB binding sites (κB sites), with the consensus being GGGRNNYYCC [8], often overlap because a κB site contains multiple G's at the 5' end and multiple C's at the 3' end with a high probability (Figure 1). For a good κB site, when the sequence window is shifted by one base in either the 5' or the 3' direction the resulting sequence is also a putative κB site. Moreover, we can have additional possibility of self-overlap because the reverse complement of a κB site is often a κB site, allowing functional binding in the opposite direction. Many computational methods do not take into account these alternative binding modes while scoring a candidate regulatory site.

Ignoring the self-overlapping nature of the motif can also complicate the interpretation of *in vitro *experiments. For example, a 3' padding sequence, starting with nucleotide C, in a gel shift experiment can form a spurious strong κB site and thus confer higher binding affinity to the experimental sequence even when the test sequence, in the context of the native promoter, makes for a very weak κB site. A computational method that naturally considers multiple binding windows and help in the design of padding sequences is useful for avoiding this problem.

The commonly used site identification methods often assume equal probability of a site everywhere in a particular window around the transcription start site (TSS) and thus fail to adjust according to the observed distribution of sites within the gene structure. Whereas proximal promoters, up to 200 bp upstream of the TSS, contain several sites, further upstream distal promoters contain fewer sites [9,10]. While identifying sites, a method should adjust its parameters to reflect this change in the density of sites with respect to the distance from the TSS.

A computational method that links to the biophysical process of transcriptional regulation can offer more biological insight than most of the current site identification methods [11,12]. Such a method can interpret statistical quantities as biophysical variables like binding energy and transcription factor concentration. It can also estimate how often a transcription factor is bound to the gene promoter, i.e. its occupancy probability. Interestingly, the use of occupancy probability as a discriminant function during site identification has a number of advantages (discussed later). One advantage of this approach is that it goes beyond just site identification and provides a measure of the importance of a site in modulating gene regulation. For example, for an activator, highly occupied sites may result in high gene expression and thus may be biologically more significant. Most methods that identify transcription factor binding sites or regulatory modules, however, leave unexplored the occupancy of the transcription factor on the promoter and its impact on gene regulation.

The lack of biophysical interpretation prevents these methods from using a natural threshold for classifying sequences into sites. They declare that a sequence is a site if its score is above an arbitrary threshold or is statistically significant compared to the score of the random background. They often have a high threshold resulting in several false negatives or a low threshold resulting in several false positives. Thus, identification of weak sites is difficult. On the contrary, saturation effects in the physical occupancy provides a natural threshold and this fact has been utilized to develop class support vector machines like QPMEME [11,12]. Alternatively, Hidden Markov Models (HMMs), when interpreted as biophysical models, overcome this problem by learning the threshold based on the transition probability to the motif state, namely the hidden state associated with a sequence pattern which is distinct from the background.

### HMM for binding site identification

HMMs, now popular in Bioinformatics for more than a decade [13], have been used in two different ways for motif identification. (1) For identification of one or more occurrences of non-overlapping sites: 'Profile HMMs' [14-16], originally designed to model protein domains, have more recently been used to identify binding sites of transcription factors, for example, of cAMP receptor protein in cyanobacterium *Anabaena *[17], liver X receptor [18] and CREB [19]. A profile HMM library was built using TRANSFAC sequences to classify transcription factors [20]. In a profile HMM, each position within a motif has three states. A match state is associated with a nucleotide being present at that position and has corresponding emission probabilities. A deletion state corresponds to absence of any nucleotide at that position. An insertion state allows for insertion of nucleotides between the current position and the next position within the motif, and has its own emission probabilities. (2) For identification of *cis*-regulatory modules (CRMs) that contain multiple sites of different types: This is usually performed using 'motif HMMs' [21-26]. In a motif HMM, the entire motif, i.e. all positions within the motif, is represented by one state. Different states correspond to different motif types (i.e. motifs associated with different transcription factors). Phylogenetic conservation has been incorporated in motif HMMs to reduce false positives [23,27-31].

Both types of HMMs, however, do not generally train their parameters properly. A profile HMM has a complicated architecture and requires a large number of parameters as a consequence. Because the number of known sites is generally small, training of a profile HMM using known sites in their native promoters (which effectively requires their simultaneous alignment) is usually not possible. Therefore, a profile HMM is generally trained using pre-aligned sites. Because the whole promoters containing the training sites are not used, transition probability to the motif *z *from background is not trained, and the relationship between transition and emission probabilities is likewise not captured. Motif HMMs, on the other hand, are more focused on identifying motifs of multiple types. But while they attempt to estimate the transition probability to the motif *z*, they generally use only the promoters containing the motifs for training *z *and thus overestimate *z*. Moreover, they usually train emission probabilities of motifs separately using pre-aligned training sites, thus ignoring the effect of *z *on emission probabilities.

Many of these HMMs use the likelihood or the Viterbi algorithm for scoring (see the Discussion section), and thus end up using an arbitrary classification threshold. Furthermore, they leave their relationship with biophysical models rather obscure and thus fail to calculate the occupancy probability. These shortcomings are in addition to their basic failure to explicitly consider the effect of multiple overlapping sites in the Viterbi method.

### Methods specifically developed for κB site identification

In an original approach to specifically identify κB sites, Udalova *et al*. developed a principal coordinate model [32-34]. They determined relative binding affinities of κB sites using experimental quantitative binding data. They selected a subset of the 256 possible variants of the fully palindromic NF-κB binding consensus sequence GGRRNNYYCC such that no variant differed from the selected sequences or their reverse complements by more than one nucleotide. They mapped these sequences to a Euclidean space and used the largest principal components for least-square linear regression of the logarithm of binding affinity in a gel shift assay or microarray. This model automatically incorporated effects of interactions between base pair positions in the binding site, its predictions were highly correlated with experimental binding data and it identified site positions responsible for differential binding of NF-κB family members consistent with crystallographic studies. However, the model's disadvantages are that it (1) requires experimental quantitative binding data of all selected sequences and (2) only includes variants of the consensus sequence. Because several known κB sites do not fit the consensus sequence [4], inclusion of all possible 10-mer variants for this model will require binding experiments with a large number of sequences, making this model infeasible. Furthermore, this model also suffers from the limitations of site identifications described above.

### Alternative interpretation of HMM as a physical binding model

Even though in the context of site identification an HMM is usually interpreted as a sequence generative model, we focus on a less familiar interpretation as a physical binding model of a transcription factor on DNA. This interpretation leads us to transform the statistical HMM model into a biophysical one. We can then determine the occupancy probability of a transcription factor on a DNA sequence, and think of the prior probability of the motif (transition probability to the motif *z*) as a measure of transcription factor concentration and the weight matrix (motif emission probabilities) as a measure of binding energies. More importantly, the biophysical model offers a principled threshold for classifying sequences into sites. The statistical mechanics model was utilized in [22] while the equivalent HMM representation is used in [23]. Although we do not know of a paper explicitly describing the equivalence of these two approaches, the work on nucleosome positioning by Segal *et al*. [5] utilizes this relationship.

An HMM is commonly used as a generative model of a spatial or time series sequence in the machine learning field [35]. In our context, the HMM consisting of two kinds of states – the background state and motif states – generates a sequence from 5' to 3'. At any position in the sequence, the HMM (1) determines the probabilities of the motif and background states at the previous position, (2) calculates the probability of either state at the current position using the transition probabilities and (3) generates the new nucleotide based upon the hidden states' emission probabilities.

We could also consider the HMM as a physical binding model that estimates the occupancy probability of a transcription factor on a particular position of a DNA sequence, i.e. how often the transcription factor is bound to that position of the DNA sequence. The relation between the two interpretations of an HMM, are as follows. In our particular HMM, the start positions of a motif state, with a particular orientation, specifies the hidden states. One could ask define the conditional probability distribution of the hidden states, or, equivalently these positions, is given the parameters and the observed sequence. It turns out that this conditional distribution is exactly the same as that of the edge positions of bound transcription factors on DNA in the statistical mechanical model. The occupancy probability at a position is the probability of finding a motif state at that position in the HMM language.

If we ignore the possibility of binding in multiple overlapping windows, then, the probability at the *j*th position of sequence *s *being the beginning of a motif is , where *W*_*j *_is the weight matrix score of the motif starting at the *j*th position of the sequence (see Methods). We claim that this quantity can be mapped to the occupancy probability, as we will see below. Thus, the two factors determining occupancy probability are (i) the transition probability to the motif *z *and (ii) the measure of distinctness of the emission probabilities of the motif from that of the background (weight matrix). Both these factors need to be high for the transcription factor to be bound to a particular position in a DNA sequence with high probability. For example, even if the weight matrix score is high, occupancy probability cannot reach one if *z *is really small. A site identification method based only on a weight matrix has no way of dealing with this interplay with *z*.

The HMM training techniques offer two advantages over the calculations made using first principles. First, the HMM Baum-Welch procedure trains the transition and emission probabilities. This produces optimized values of *z *and the weight matrix, which are essential for an accurate estimation of occupancy probability. Secondly, as mentioned above, the HMM training procedure also calculates an intermediate variable called the gamma (*γ*) variable. The occupancy probability at a particular position is given simply by the gamma variable of the motif state at that position: , where  and  are the gamma values of the motif and background states at that position, respectively (see Additional file 1). Thus, the calculation of occupancy probability in a new sequence requires a simple extension of the scoring procedure in which the gamma variable is computed just like during the training procedure.

In a biophysical model, based on thermodynamics principles, occupancy probability can be written as , where [*P*] is the concentration of a free transcription factor at equilibrium, *E*_*j*_(*s*) is the binding energy at position *j*,  where *K*_*b *_is the Boltzmann's constant, and *T *is the absolute temperature [11]. A comparison of this equation with the equation of probability obtained from the HMM gives us *W*_*j *_= - *βE*_*j*_(*s*) and *z *= [*P*]. Thus, the weight matrix represents binding energy [1]. In addition, the transition probability to the motif *z *corresponds to the free transcription factor concentration. As the transcription factor concentration increases, the transition probability to the motif increases and we expect higher occupancy by the transcription factor on the DNA.

One main focus of this study is to determine a good threshold for the classification of sequences into binding sites. Use of an arbitrary threshold while using a purely statistical quantity as a discriminant function is prone to a high number of false positives or false negatives. A good discriminant function has a physical interpretation, it relates to the biological significance of a sequence and it offers a natural threshold for classification. Moreover, the classification method needs to be able define the above threshold based on training sequences.

We use occupancy probability as a discriminant function to classify sequences into sites because it has the following benefits. First, unlike a purely statistical entity, it has a straightforward physical interpretation. A transcription factor's occupancy on the promoter determines gene expression. Second, highly occupied binding sites may be physiologically more significant. Thus, occupancy probability not only helps in classifying sequences as binding sites but also offers insight into their influence on gene expression. Third, occupancy probability has a "natural" threshold at 0.5 due to its Fermi-Dirac distribution with respect to binding energy [11]. Sequences with binding energy less than the chemical potential have occupancy probability greater than 0.5 and hence can be classified as sites. Because of our focus on occupancy, we call this model OHMM (**O**ccupancy via **H**idden **M**arkov **M**odel).

The greatest benefit of the transformation of an HMM to a biophysical model is that it enables the HMM to learn the threshold for classification of sequences into sites in a principled way. We have seen above that the occupancy probability depends upon the transition probability to the motif *z *and the weight matrix, both of which are trained by an HMM. Thus, when an HMM uses occupancy probability as a discriminant function, it learns the natural threshold based on the training sequences. Because of this accurate estimation of the threshold, an HMM is expected to identify weak sites much more accurately with a fewer false positives.

**Figure 2:**
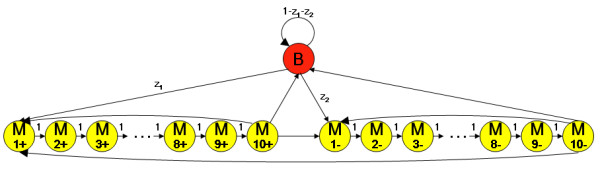
**HMM hidden states for κB sites**. OHMM consists of 21 states. The background state is colored red and designated by B. Each of the 20 motif states corresponds to each of the ten positions within the κB motif on the two DNA strands. The motif states are colored yellow and designated using M, the position within the motif and the strand. The emission probabilities of the motif states on the two strands are flipped from 5' to 3' so as to represent identical binding irrespective of the motif strand. Non-zero transition probabilities between states are represented by black arrows and their values are shown.

In summary, we describe an HMM, applied to NF-κB, that identifies overlapping sites, learns the threshold in a principled manner by training emission probabilities using known sites in their native promoters and training transition probabilities using promoters in an entire genome, estimates the transcription factor's occupancy probability, and adjusts parameters reflecting the distance from the TSS while scoring. The structure of hidden states in OHMM is indicated in Figure 2. We demonstrate that the occupancy probability predicted by OHMM correlates well with the binding affinity in gel shift experiments. High evolutionary conservation scores and enrichment in experimentally regulated genes suggest that the predicted sites might be functional.

## Results

### HMM training

Training OHMM consists of estimating two sets of parameters: (1) the emission probabilities of each motif state (motif profile) and the background, and (2) the transition probabilities that depend upon the transition probability to the motif (*z*). While an HMM needs to be trained using site-rich sequences to learn the motif profile, training on random sequences is required to learn *z *reflecting the site density in the promoters of all genes in the human genome. We therefore estimated HMM parameters in two steps. We first trained all HMM parameters using site-rich sequences and thus learned the emission probabilities. We then fixed the emission probabilities and trained the transition probabilities using randomly selected human promoters to estimate *z *separately for the TSS-800:TSS (upstream 800 bp) and TSS:TSS+100 (downstream 100 bp) regions, where TSS is the transcription start site. The reason for separate *z *estimation in these two types of regions is that κB site density is different upstream and downstream of the TSS.

### Training the emission probabilities

**Figure 3:**
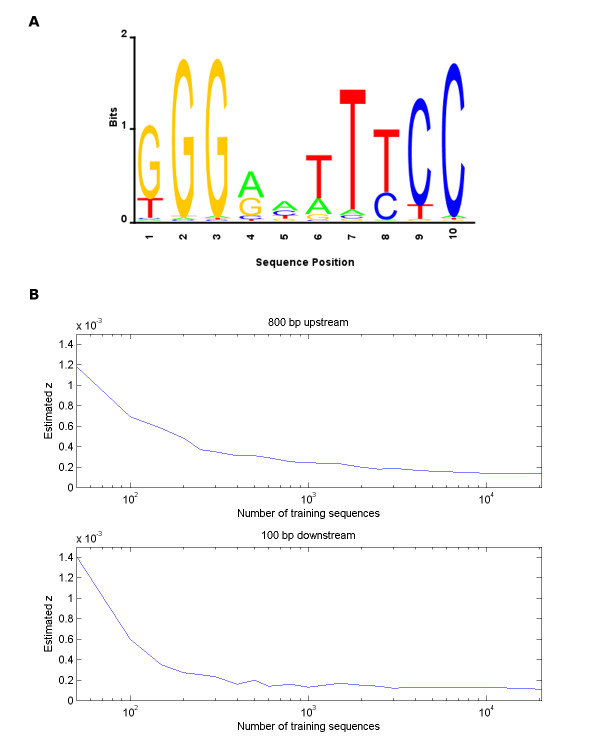
**Trained HMM Parameters**. A. Sequence logo of the motif profile of the HMM trained on 50 bp sequences each consisting of a known κB site and surrounding region (surround-50 HMM) with initial transition probability to the motif (*z*) equal to 0.02. B. The estimated transition probability to the motif (*z*) for upstream 800 bp and downstream 100 bp regions with respect to the transcription start site (TSS) as the number of randomly selected training genes increases. The estimated *z *stabilizes after the addition of a few thousand genes. Each training set contains all sequences with known κB sites in the relevant region (20 and 4 known sites for the upstream 800 bp and downstream 100 bp regions, respectively).

In the first training step, we used two types of site-rich sequences of different lengths and various initial *z*'s to train all HMM parameters and determine the emission probabilities to be used in further analysis. Trained motif profiles of "TSS-n HMMs," i.e. HMMs trained on promoters consisting of n bases upstream of the TSS of human genes known to contain a κB site, appear similar to the background emission probabilities regardless of the promoter length and initial *z*. On the other hand, trained motif profiles of "surround-50" or "surround-100 HMMs," i.e. HMMs trained on 50 or 100 bp sequences each consisting of a known κB site and surrounding region, with a reasonable initial *z *(between 0.0001 and 0.01), are distinct from the background (see Figure 3A, Additional file 2). They are also distinct from the initial motif profile, as their symmetrical Kullback-Leibler (KL) divergences (defined as , where *P*_*i *_and *Q*_*i *_are the emission probability distributions of the *i*th motif positions of motif profiles *P *and *Q*, ℓ is the motif length and *D*_*KL *_is the log e-based KL divergence) with respect to the initial motif profile are high (0.49 and 0.5, respectively; in comparison, the KL divergences between the initial motif profile and 100 multinomial distributions simulated from the initial motif profile have a normal distribution with mean 0.0015 and standard deviation 0.00038). The trained motif profiles are slightly weaker than the initial motif profile, i.e. more similar to the background. In a surround-50 or surround-100 HMM, any initial *z *between 0.0001 and 0.01 results in the same trained motif profile, indicating that perhaps a local optimum is reached. Trained motif profiles of surround-200 HMMs, however, appear more and more like the background as the initial *z *increases above 0.001. Trained motif profiles of surround-400 HMMs appear similar to the background regardless of the initial *z*. We used the trained motif profile of the surround-50 HMM for further analysis (Figure 3A).

### Estimation of the transition probability to the motif

In the second training step, we estimated *z *by training the transition probabilities of the above surround-50 HMM using random sequences while keeping its emission probabilities constant. We estimated *z *separately for the upstream 800 bp and downstream 100 bp regions. In each case, we determined human promoters with known κB sites in that region, progressively added randomly selected human promoters to the list and estimated *z*. As expected, the *z *estimate decreased with the addition of random sequences until it stabilized after the addition of a few thousand promoters (see Figure 3B). The estimated *z*'s for the upstream 800 bp and downstream 100 bp regions of all genes in the human genome are 0.00017 and 0.00012, respectively.

### Effect of the nature and length of training sequences, initial transition probability to the motif (initial *z*) and training of motif profile on trained *z*

**Figure 4:**
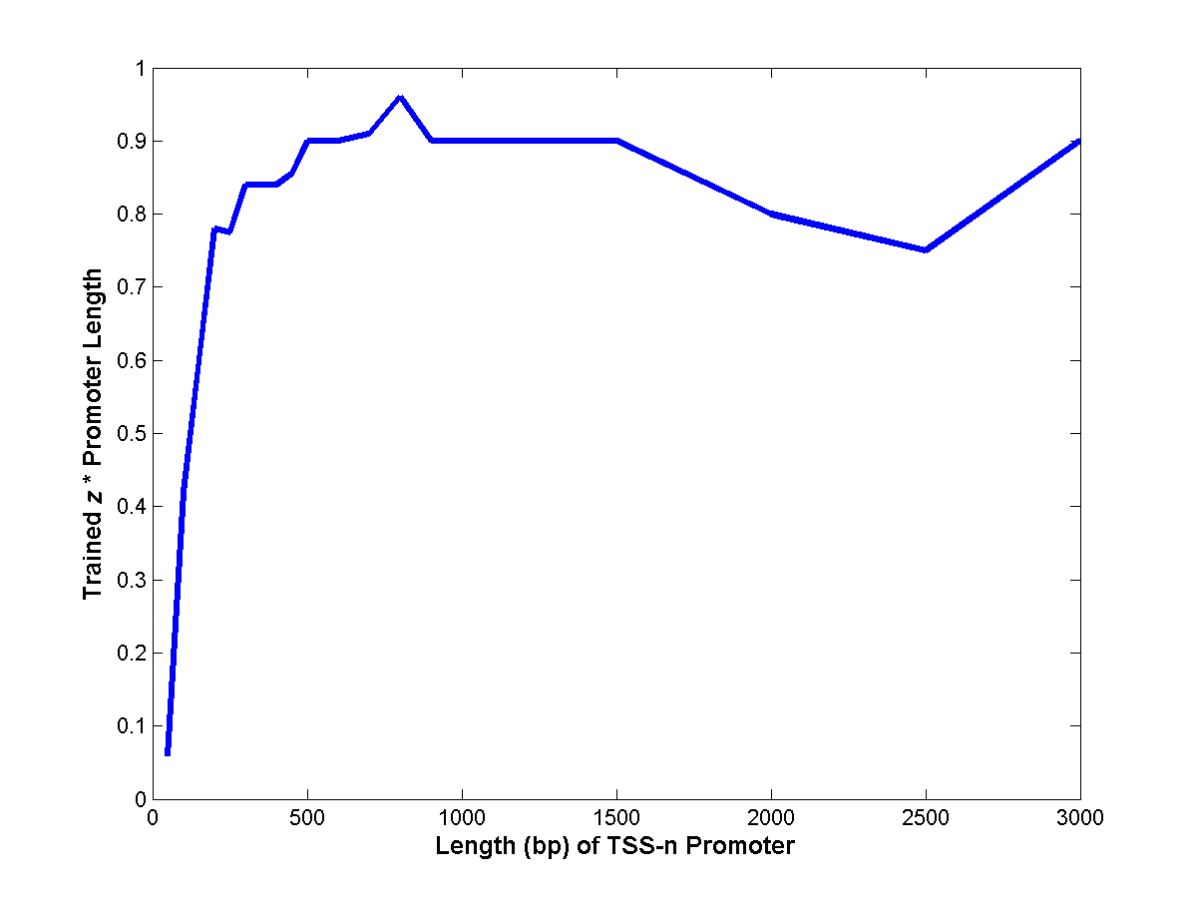
**Trained *z *is inversely proportional to the length of the training promoter**. HMMs were trained on TSS-n promoters keeping the initial motif profile fixed. The transition probability to the motif (*z*) is inversely proportional to the training promoters' length in the range between 500–3000 bp and hence z* is constant around 0.9. This quantity drops slightly between 500 to 200 bp and then substantially after 200 bp due to the lack of κBsites in the shorter training promoters.

We examined the effect of the nature and the length of training sequences, the initial *z *(transition probability to the motif) and the effect of the motif profile on trained *z*. Motif profile was kept fixed in this investigation to isolate the effect on *z*. When trained on TSS-n promoters and the initial motif profile, *z *is inversely proportional to the training promoters' length in the range between 500–3000 bp. Hence, the quantity *z *is relatively constant at ~0.9 (Figure 4). It drops slightly between 500 to 200 bp and then substantially after 200 bp due to the lack of κB sites in the shorter training promoters. When trained on surround-n promoters and the initial motif profile, the trained *z *is again inversely correlated to the training promoters' length, but the above quantity is higher at ~1.8 (*z *= 0.0347, 0.0175 and 0.0087 when n = 50, 100 and 200, respectively). This quantity is even higher at ~1.9 when a slightly weaker motif profile corresponding to a surround-n HMM is used (and kept fixed) instead of the initial motif profile (*z *= 0.0363 and 0.02 when n = 50 and 100, respectively). The initial *z *of TSS-n or surround-n HMMs in the range between 0.0001 and 0.1 does not affect the trained *z*, probably because a global optimum is reached after a few expectation maximization (EM) iterations during training.

### Varying *z *with respect to the distance from the TSS while scoring

As mentioned in the background, site density decreases in locations that are increasingly further upstream of the TSS [9,10]. Also, site density at a location in a sequence is proportional to the transition probability to the motif (*z*) at that location. Therefore, while predicting sites in upstream 800 bp regions, we modeled *z *using an exponential functional form such that a sequence close to the TSS had a higher site density than a sequence further upstream. The maximum likelihood estimate of the mean distance of κB sites upstream of the TSS was 170 bases. Interestingly, the estimate of the mean distance using the median was about the same (169 bases). By equating the expressions for the site density per promoter when *z *had a uniform or exponential function form, the scale factor in the exponential form was z_0 _= 0.137 for the uniform *z *= 0.00017 obtained in training (see Methods for details). A different transition probability matrix was generated for each upstream position based on the value of *z *at that position and was used to calculate occupancy probability. On the other hand, when identifying κB sites in the downstream 100 bp regions, we used location-independent transition probabilities based on the uniform *z *of 0.00012 obtained in training. This choice was made due to the paucity of evidence of positional dependence of site density in these regions.

### OHMM performs better than a WM

**Figure 5:**
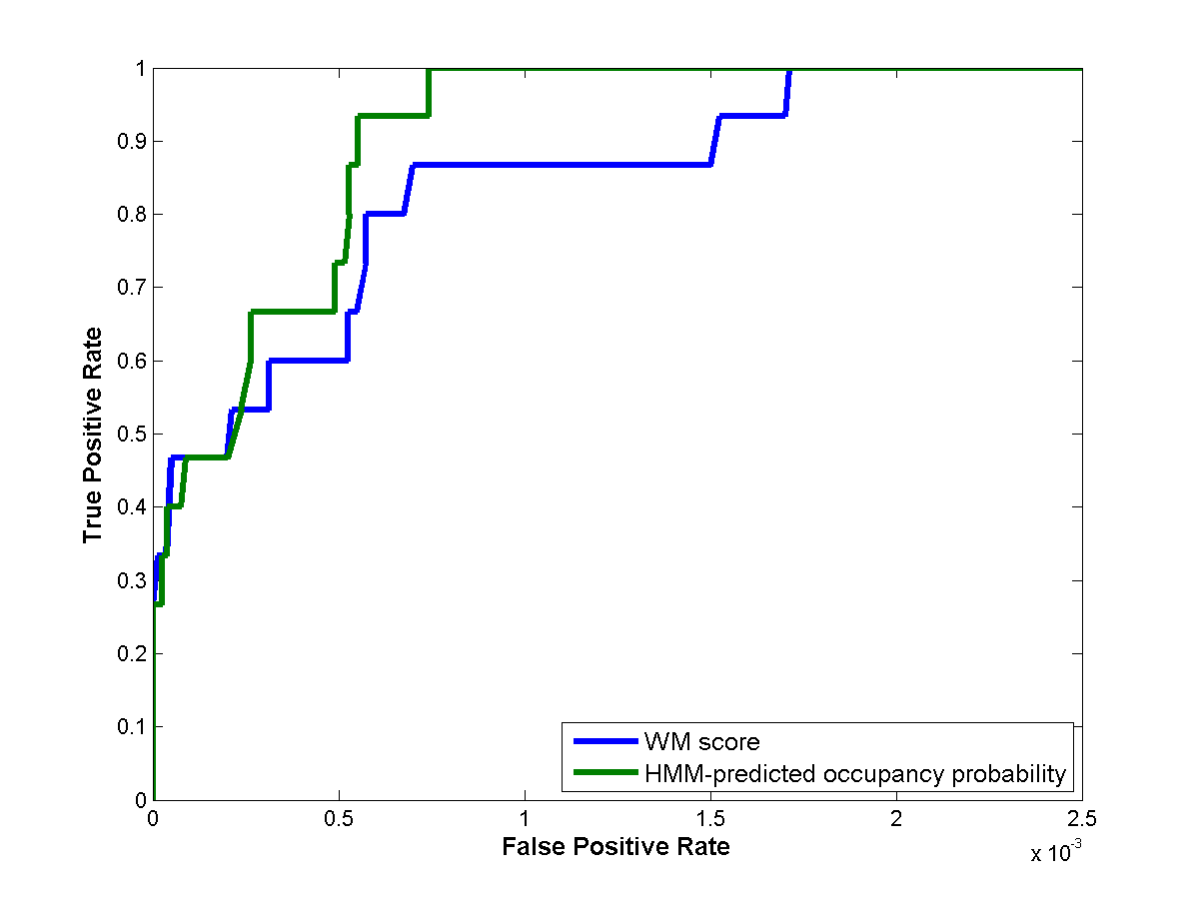
**ROC analysis shows that OHMM performs better than a weight matrix**. The performances of the HMM and the weight matrix (WM) are represented by the green and the blue curves, respectively. Whereas the HMM and the WM perform similarly for strong sites, the HMM is more accurate in identifying weak sites. The positive examples consist of the 36 known human κB sites present in upstream 800 bp regions (in their native promoters), and the negative examples consist of all 10-mers in the upstream 800 bp regions in 100 randomly selected human genes as described in the text. Leave-one-out cross-validation was performed. ROC: Receiver Operating Characteristic curve.

The ROC analysis shows that OHMM performs better than a weight matrix (WM) (Figure 5). While both the HMM and the WM are highly accurate when identifying strong sites, the HMM is more accurate in identifying weak sites. The segregation of weak sites from site-like sequences is quite difficult due to degeneracy and provides a crucial test. In this respect, our model far outperforms the WM. We believe that this superior performance of OHMM is the result of training the threshold in a principled manner to minimize false positives and false negatives. The positive examples consist of the 36 known human κB sites present in upstream 800 bp regions (in their native promoters) and the negative examples consist of all 10-mers in the upstream 800 bp regions in 100 randomly selected human genes as described in the Methods section. Leave-one-out cross-validation was performed in this analysis.

### Many κB sites predicted by OHMM are evolutionarily conserved and regulated after NF-κB over-expression

**Figure 6:**
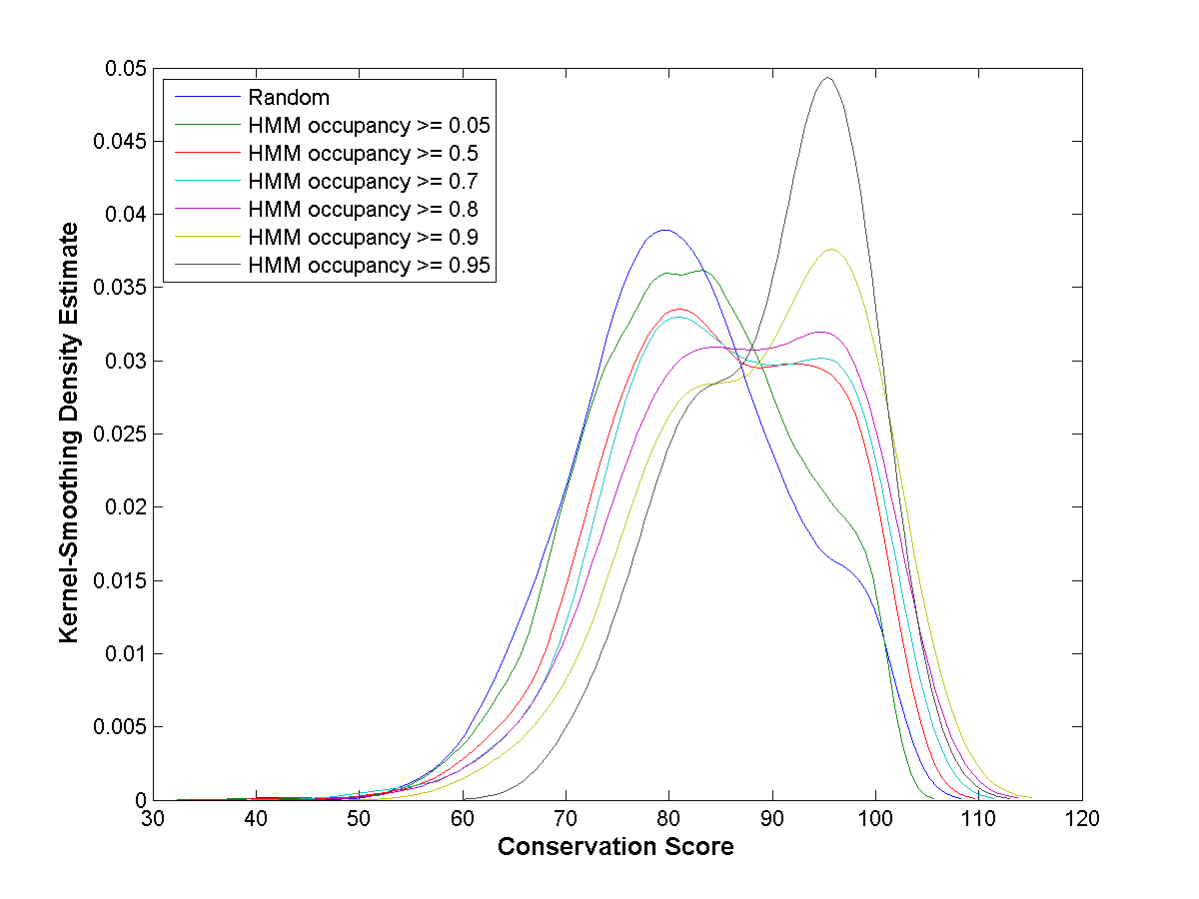
**κB sites with greater HMM occupancy probability are conserved better**. Each curve represents the kernel-smoothing density estimate of the evolutionary conservation scores of a set of κB sites. Each set consists of κB sites predicted by OHMM to have occupancy probability above a threshold shown in the legend. The "random" set consists of 1000 10-tuples randomly selected from the human promoters. Conservation scores of κB sites predicted by OHMM are higher than those of the random sequences. Moreover, κB sites with higher HMM occupancy probability have higher conservation scores. Conservation scores and kernel-smoothing density estimates were calculated as described in the Methods section.

We predicted κB sites in all gene promoters in the human genome and calculated their occupancy probabilities. Two types of data suggest that they may be functional sites. First, evolutionary conservation scores of κB sites predicted by OHMM are higher than those of 1000 10-tuples randomly selected from human promoters, and κB sites with higher HMM occupancy probability have higher evolutionary conservation scores (Figure 6). Secondly, the chicken genes regulated by over-expressed NF-κB proteins in a microarray experiment [36] and their human orthologs are enriched with κB sites predicted by OHMM (see Additional file 3). Notably, genes regulated in a higher number of comparisons are more enriched with HMM-predicted sites. Also, OHMM predicted more κB sites per regulated gene among genes predicted to contain κB sites probably because true NF-κB targets contain multiple sites. Interestingly, human orthologs of regulated chicken genes are more enriched with predicted NF-κB targets than the chicken genes themselves probably due to the availability of higher quality sequences for humans. In this experiment, seven NF-κB proteins from different species were over-expressed in chicken DT40 pre-B cell lines, and regulated genes were identified by comparing the expression level for each experimental condition against the control.

### Quantitative measure of biological significance

**Figure 7:**
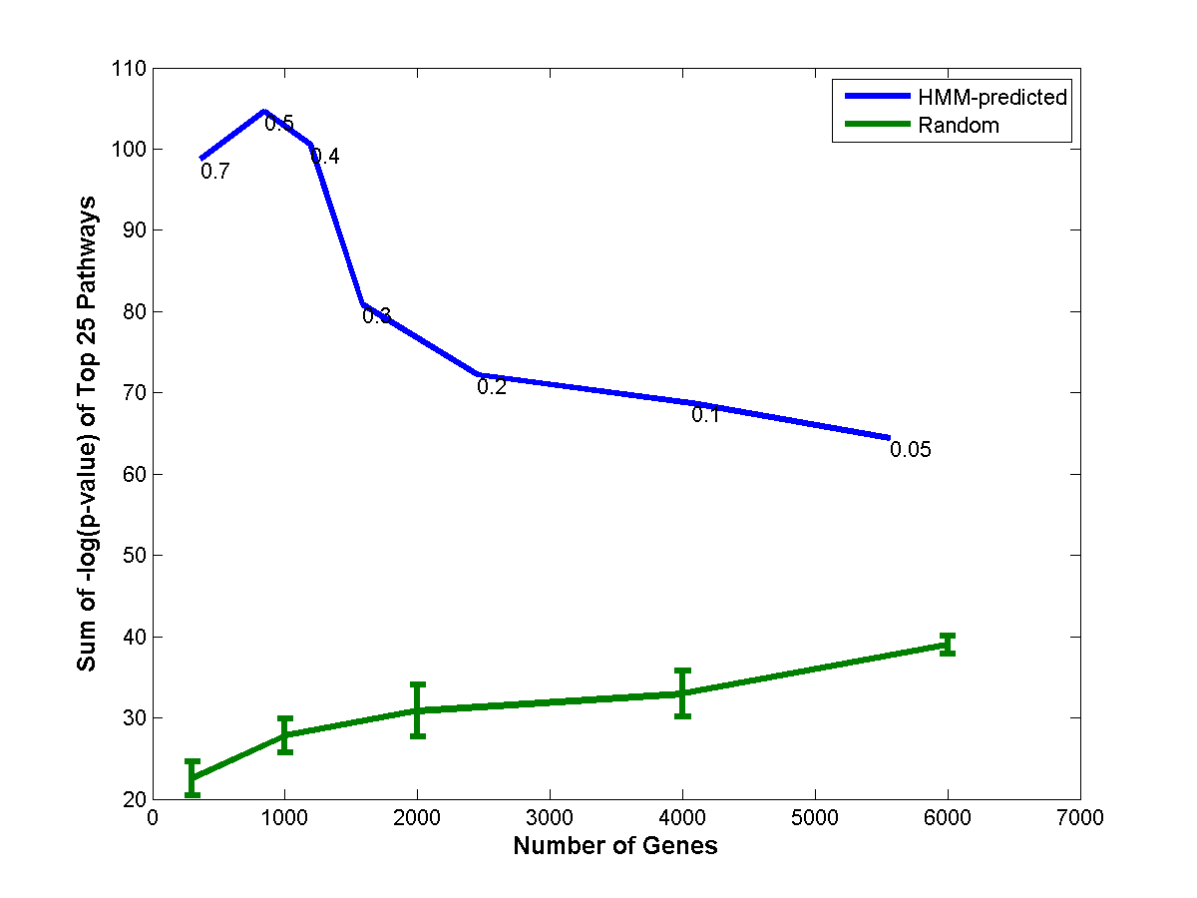
**Biological significance of predicted target gene sets using pathway analysis**. Biological significance is shown with the help of the pathways enriched in the κB target gene sets predicted by the HMM at various thresholds. The y-axis shows the sum of the negative logarithm of the p-values of the top 25 enriched pathways. Gene sets predicted by the HMM are biologically significant as compared to randomly selected genes. They show a peak at the threshold occupancy probability of 0.5 (~800 genes). The thresholds used for obtaining the gene sets for the pathway analysis (occupancy probability threshold between 0.05 and 0.7) are indicated. HMM-predicted gene sets and randomly selected gene sets are indicated by blue and green curves, respectively. Only about 50–70% of the genes in each gene set are available for pathway analysis because the rest of the genes are not adequately annotated. The numbers in the figure, however, correspond to the number of genes in the entire gene sets.

With the aim of determining the biological significance of the sets of κB target genes predicted by OHMM at the various thresholds, we used pathway analysis to discover the pathways enriched in these κB target genes (Figure 7). The sum of the negative logarithm of the p-values of the top 25 enriched pathways was used as the measure of biological significance. It is useful to note that only about 50–70% of the genes in each gene set are available for pathway analysis because the rest of the genes are not adequately annotated. The numbers in Figure 7, however, correspond to the number of genes in the entire gene sets. The gene sets predicted by the HMM at various occupancy probability thresholds are much more biologically significant than randomly selected genes. Moreover, the biological significance reaches a peak at the occupancy probability threshold of 0.5 (corresponding to ~800 genes). This implies that the gene sets corresponding to the thresholds greater than 0.5 have many false negatives, because of which these gene sets do not have enough key target genes to attain high significance. On the contrary, the gene sets corresponding to the thresholds less than 0.5 have many more false positives which dilute these sets and lower their biological significance. Thus, the HMM appears to identify sites most accurately in a short window around the threshold of 0.5. This observation ties excellently with our justification for training the HMM threshold and the use of occupancy probability as the discriminant function.

### Correlation with gel shift experiments

A common way to measure affinity of a TF for a particular DNA sequence is the gel shift assay, some times also called electrophoretic mobility shift assay or EMSA. In this assay, oligonucleotides (usually radio-labeled) with the particular DNA sequence is mixed with the TF at a certain concentration. At equilibrium, a certain fraction of these oligonulcotides will be bound to the TF. The oligonucleotides are then passed through a polymer gel under electric field. DNA bound to TF moves at a different rate (usually slower) than the unbound ones, producing a shift between the two bands. The relative amount of DNA in each of these two bands could be measured, usually by scanning for radioactivity, allowing us to calculate the occupancy probability.

**Figure 8:**
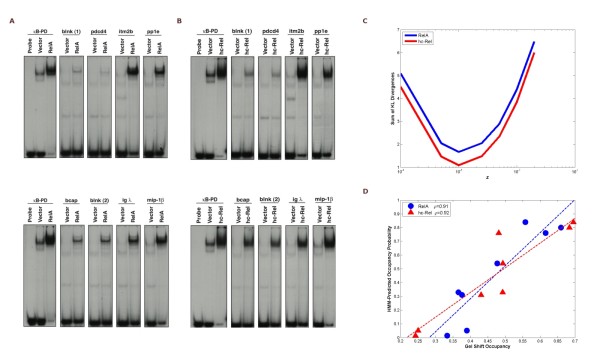
***In vitro *binding affinity of NF-κB's RelA and c-Rel proteins to κB sites correlates well with HMM-predicted binding occupancy probability**. Gel shift assays with extracts from 293T cells transiently transfected with either CMV-hRelA (A), CMV-hc-Rel (B) or empty CMV vector as control (vector) and radiolabeled double-stranded oligonucleotide probes containing the predicted NF-κB sites derived from chicken *blnk *site 1 or site 2, *pdcd4*, *itm2b*, *pp1e, bcap*, *igλ*, or *mip-1β*, or a palindromic NF-κB DNA site as control (κB-PD). Reactions containing the κB-PD probe alone, in absence of cell extract, were loaded as control (probe). DNA/protein complexes were resolved from unbound DNA probes in native 5% polyacrylamide gels. (C) shows the sum of Kullback-Leibler (KL) divergences of the HMM-predicted occupancy probabilities of the above sequences (in the gel shift constructs) with their binding affinities in the gel shift experiments, as a function of the transition probability to the motif *z*. The sum of the KL divergences is minimum at *z *equal to 0.001 for both NF-κB proteins. (D) shows the correlation between the gel shift binding affinities of the above sequences and their occupancy probabilities predicted by the HMM at *z *equal to 0.001. The correlation coefficients are 0.91 and 0.92 in case of RelA and c-Rel, respectively. The dashed lines in (D) are linear least square fits.

We performed gel shift experiments using double-stranded radio-labeled oligonucleotide probes containing 10-mers derived from several chicken promoters to determine if OHMM predicted the occupancy probability accurately (Figure 8). We performed the experiments for two NF-κB family members: RelA and c-Rel. The transition probability to the motif (*z*) in a gel shift experiment is higher than in the cellular context due to higher protein concentration in the gel (see the discussion below). However, because it is difficult to know the protein concentration in the gel, we estimated *z *for both RelA and c-Rel (see Methods). The estimated *z *is 0.001. Occupancy probabilities predicted by the HMM correlate well with the binding affinities of RelA and c-Rel proteins to these sites at this *z *(correlation coefficients of 0.91 and 0.92, respectively).

### Effect of *z *on HMM-predicted occupancy probability

**Figure 9:**
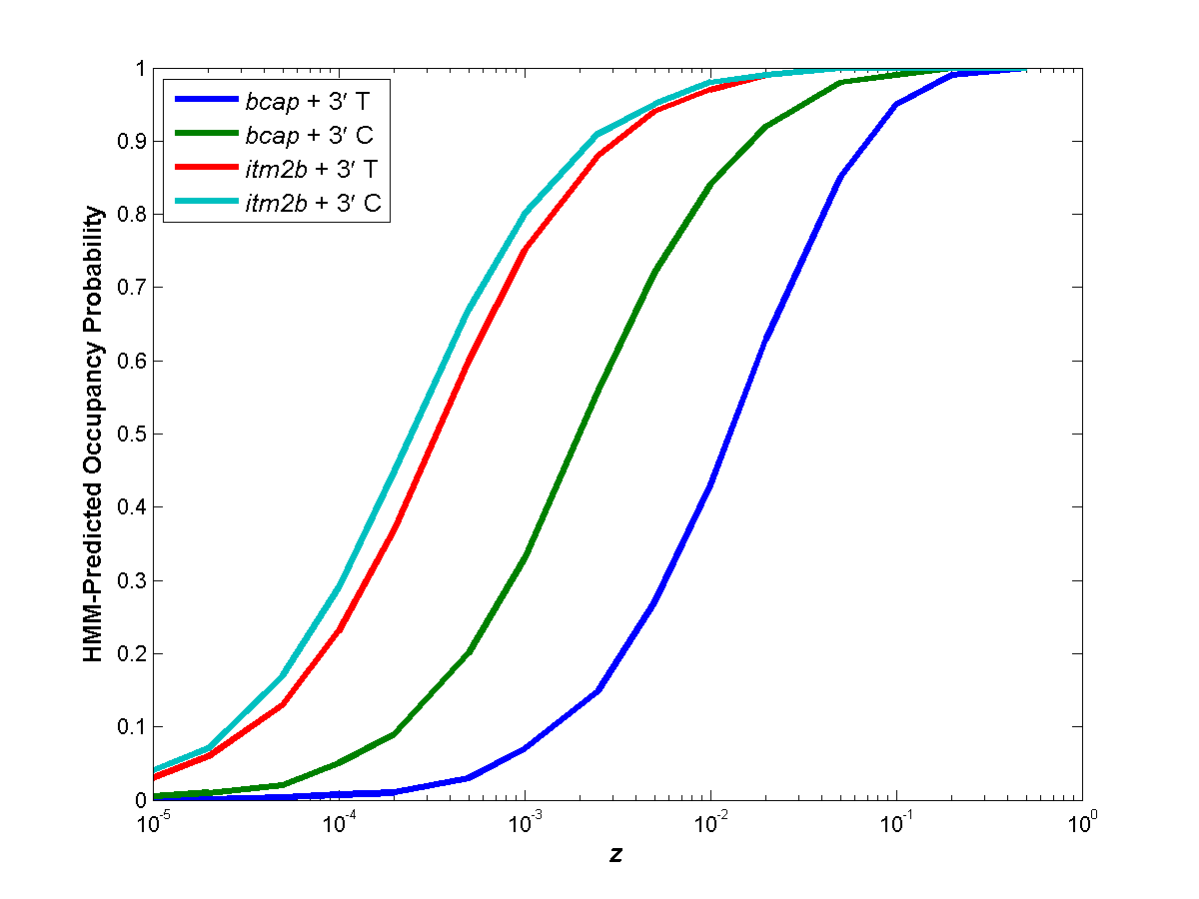
**Occupancy probability increases sigmoidally with respect to *z*, is greater for stronger κB sites and depends upon the padding sequences in the case of self-overlapping binding motifs**. Occupancy probability of the *bcap *and *itm2b *oligonucleotides used in the gel shift experiment, with either a C or a T at the beginning of the 3' padding sequence, was predicted using an HMM with different *z*'s. The HMM's motif profile was the same in all instances. The predicted occupancy probability rises as a sigmoidal function of *z*. The occupancy probability of the stronger κB site (*itm2b *vs. *bcap*) saturates at lower *z*, and therefore the occupancy probability of the stronger site is greater at a particular *z*. Moreover, the occupancy probability of oligonucleotides is greater when the 3' padding sequence begins with a C (resulting in a stronger spurious site) than a T.

We plotted the HMM-predicted occupancy probability with respect to *z *while keeping the same motif profile (Figure 9). Three characteristics of the dependence between *z *and occupancy probability stand out: (1) Occupancy probability increases sigmoidally and then saturates as *z *increases. (2) Occupancy probability of a stronger site (e.g. *itm2b *vs. *bcap *κB site in Figure 9) saturates at lower *z*, and therefore occupancy probability of the stronger site is greater at a particular *z*. (3) Occupancy probability is influenced by surrounding sequences due to the formation of spurious sites (e.g. it is higher when the 3' padding sequence of a κB site in a gel shift construct starts with a C than with a T).

### HMM reveals the importance of selection of padding sequences of self-overlapping motifs

The occupancy probabilities calculated using the sites in their native chicken promoters did not correlate as well with the experimental binding affinities as those calculated in the previous section using the sites and their padding sequences in the gel shift construct. When we observed that the difference was due to a C in the padding sequence 3' of the predicted κB sites in the oligonucleotides used for gel shift, we performed a systematic combinatorial analysis using HMM to determine the padding sequences that did not form spurious overlapping binding sites and hence affected native binding the least. We found that the use of the padding sequences in the above experiment (the 5' padding sequence is GATCTGAATTCGT and the 3' padding sequence is CACCTCTCCTTA) may misrepresent NF-κB binding. The predicted occupancy probabilities suggest that a gel shift oligonucleotide containing an A 5' to the 10-mer and a T 3' to the 10-mer in the padding sequence has the least chance of forming spurious binding sites (e.g. A*GGGAATTCCC*T, where the 10-mer is shown in italics). Any other nucleotide forms a spurious site shifted one position from the 10-mer, and in some cases may even change the binding occupancy by more than 50%. Any of the C, G or T in the 5' end creates a site beginning with CGG..., GGG... or TGG.... An A, C or G in the 3' end creates a site on the opposite strand beginning with TGG..., GGG... or CGG.... In addition, a C at the 3'end also creates a site on the same strand ending with ...CCC.

### Identification of NF-κB targets in the human genome

As mentioned above, we predicted κB sites in the upstream 800 bp and downstream 100 bp regions (with respect to the TSS) of all genes in the human genome using the exponential and uniform *z *respectively (see Additional file 4). Genes containing at least one κB site with predicted occupancy probability greater than or equal to 0.5 are designated as putative direct targets of NF-κB. We also identified cellular pathways, biological functions and diseases in which our predicted NF-κB targets were over-represented. As expected, we found many known NF-κB target genes with roles in B or T cell receptor signaling, NF-κB signaling, cytokine and chemokine signaling, antigen presentation, acute phase response, or in death receptor and apoptosis signaling among others (Table 1; see Additional file 5). Importantly, OHMM also pinpointed several novel candidate NF-κB targets in these and other pathways that have not yet been described to be regulated by NF-κB [37,38]. This is exemplified by identification of κB sites for DIABLO, which potentiates some forms of apoptosis, and for the TRAF family-associated NF-κB activator TANK that inhibits NF-κB activation and enhances apoptosis by activating cell death signals and inhibiting survival signals. Although it remains to be determined if these and other genes identified using OHMM are genuine NF-κB transcriptional targets, if confirmed, these could yield important new insights into the less well-characterized but nonetheless important pro-apoptotic activity of NF-κB that has been observed in response to certain stimuli and in certain cells [39,40].

**Table 1:** Enriched pathways, functions and diseases

Pathway/Function/Disease	Gene Symbols
NF-κB Signaling	NFKB2*, CD40*, IL1F9, IKBKB, RRAS, TNFAIP3*, BCL3*, TLR7, TRAF5, NFKBIB, NFKB1*, LTA*, PIK3C3, NFKBIA*, RELB*, BTRC, PIK3R2, ZAP70, TRAF3, IL1RN*, PLCG2, MAP3K8

Glucocorticoid Receptor Signaling	VCAM1*, ICAM1*, MED1, SMAD3, IKBKB, RRAS, MAPK12, BCL3*, IL13*, CCL5*, NFKBIB, NFKB1*, IL8*, PIK3C3, NFKBIA*, NR3C1*, STAT1, CXCL3*, CREB1, PIK3R2, JAK3, SELE*, IL1RN*, IL6*

Antigen Presentation Pathway	B2M*, PSMB9*, HLA-A, CD74, HLA-B*, HLA-DQA1, TAPBP*

Acute Phase Response Signaling	SAA1*, IL1F9, RBP1, IKBKB, RRAS, MAPK12, BCL3*, SERPINA3*, NFKBIB, CFB*, NFKBIA*, NR3C1*, PIK3R2, NOLC1, SAA2*, SOCS2, IL1RN*, IL6*

B Cell Receptor Signaling	IKBKB, RRAS, MAPK12, BCL3*, NFKBIB, CALML5, NFATC1, PTPN6, NFKBIA*, PIK3C3, CREB1, MAP3K11, PIK3R2, PLCG2, MAP3K8

Death Receptor Signaling	NFKBIA*, BIRC3, DIABLO, IKBKB, BCL3*, TANK, NFKBIB, TNFSF15*

Apoptosis Signaling	NFKBIA*, BIRC3, DIABLO, IKBKB, RRAS, BCL3*, MAPK6, TP53*, NFKBIB, RPS6KA1, PLCG2, MAP3K8

Cell Cycle: G1/S Checkpoint Regulation	BTRC, SMAD3, SIN3A, TP53*, HDAC8, E2F6

Chemokine Signaling	CCL4*, RRAS, CCR3, MAPK12, CCL5*, PLCG2, CALML5

T Cell Receptor Signaling	NFATC1, PIK3C3, NFKBIA*, IKBKB, RRAS, PIK3R2, ZAP70, CALML5

Notch Signaling	DLL1, NOTCH2, RBPJ, MAML2

P53 Signaling	BBC3, PIK3C3, SIRT1, PPP1R13B, MED1, PIK3R2, TP53*

Xenobiotic Metabolism Signaling	IL4I1, SULT1C2, MED1, RRAS, MAPK12, NFKB1*, NFKB2*, GSTP1*, PIK3C3, PPP2CB, ALDH3B2, EIF2AK3, PIK3R2, NFE2L2, IL6*, IL1RN*, GSTA5

Neurotrophin/TRK Signaling	PIK3C3, CREB1, RRAS, PIK3R2, RPS6KA1

Protein Ubiquitination Pathway	UBE2H, UBE2D3, B2M*, UBE2M*, BIRC3, BTRC, PSMB9*, HLA-A, HLA-B*

Skeletal and Muscle Development and Function	CD40*, CSF1*, CXCL11*, DLL1, IKBKB, IL6*, IL13*, IL1RN*, MED1, NFATC1, NFKB1*, NFKB2*, NFKBIA*, RBPJ, SMAD3, STAT1, VCAM1*, WNT10B*

Infection of Virus	CCL4*, CCL5*, CLEC4M, DEFA1, ICAM1*, IL13*, IRF8, XPO1

Cancer	ACACA, AIM2, B2M*, BBC3, BCL2L10, BIRC3, BTRC, C6ORF66, CARD8, CD40*, CREB1, CTGF, CYLD, DBC1, DIABLO, DLL1, DPP4, DUT, EGR2, EIF2AK3, GNB1, GNB2L1*, HINT1, HUWE1, IER3*, IFNB1*, IGFBP6, IL6*, IL8*, IL13*, IL1RN*, IRF1*, IRF8, ITGA5, LCN2*, LTA*, LTB*, MAML2, MAP3K11, MAPK12, MEN1, MIA, MSX1, MYB*, NFKB1*, NFKB2*, NFKBIA*, NFKBIZ, NR3C1*, OAS3, PLCG2, PPP1R13B, PPP5C*, PTPN6, RBM17, REL*, RHOC, RPS6KA1, RUNX1T1, SMPD2, STAT1, THOC1, TNFAIP3*, TNFSF13, TP53*, TRAF3, TWIST1*

Rheumatoid Arthritis	ACAN, ACTA1, ADAMTS7, B2M*, BLR1*, CARD8, CCL1*, CCL4*, CCL5*, CCL19*, CD40*, CD69*, CD70, CD74, CD83*, CD86*, CD274*, CFB*, CXCL1*, CXCL2*, CXCL3*, CXCL5*, CXCL6*, CXCL10*, DEFA1, DPP4, GP1BA, HLA-A, HLA-DQA1, HPRT1, ICAM1*, IFNB1*, IL6*, IL8*, IL13*, LTA*, LTB*, MAPK12, NFKB1*, NFKBIA*, NR3C1*, PSMB9*, SAA1*, SAA2*, TNFAIP3*, TNFRSF13B, TNFSF15*, TP53*, TPM2, VIM*, WNT10B*

Experimental Autoimmune Encephalomyelitis	B2M*, CD40*, CD86*, CXCL10*, DPP4, HLA-DQA1, IFNB1*, IKBKB, IL6*, LTA*, LTB*, NR3C1*, REL*, STAT1

Selected cellular pathways, biological functions and diseases in which our predicted NF-κB targets were over-represented are shown. The associated predicted NF-κB targets are represented by official human gene symbols. Genes containing κB sites with predicted occupancy probability greater than 0.5 were used in this analysis. Please see Additional file 5 for the complete list. Genes known in the literature to be regulated by NF-κB (although not necessarily directly) [38] are denoted with *.

Similarly, OHMM pinpointed κB sites for IKBKB (commonly known as IKKBeta or IKK2), which is the key NF-κB activating kinase in the canonical NF-κB signaling pathway (see the discussion below), as well as for Toll-like receptor 7 (TLR7) that serves as a coreceptor for RNA-associated autoantigens and participates in the innate immune response to microbial agents, potentially extending the list of NF-κB-regulated TLRs (TLR2, TLR9). If validated as genuine NF-κB targets, identification of IKKBeta and TLR7 by OHMM may have uncovered novel positive feedback loops for amplification of NF-κB signaling.

Our analysis suggests that NF-κB could play new roles in some of the pathways in which it is already known to participate, as seen in the protein ubiquitination pathway that can either regulate protein activation/function or target proteins for proteolytic degradation via the proteasome. For instance, while it is known that NF-κB activates the expression of deubiquitinating enzymes CYLD and A20 (TNFAIP3) that negatively regulate NF-κB signaling [41-44] as well as expression of the proteasome subunit LMP2 (PSMB9) and proteasome activators PA28-alpha and PA28-beta [45-47], OHMM identified the E2 ubiquitin-conjugating enzymes UBE2H, UBE2D3 and UBE2M that promote protein ubiquitination, as putative NF-κB targets. Incidentally, UBE2M was previously observed in microarray studies to be induced by Epstein-Barr virus that leads to increased NF-κB activity, although it remains to be determined if UBE2M is directly controlled by NF-κB [48].

Interestingly, our analysis also pinpointed κB sites in BTRC (better known as beta-TrCP). BTRC is the substrate recognition component of a SKP1-CUL1-F- box protein (SCF) E3 ubiquitin ligase complex that mediates ubiquitination and subsequent proteasomal degradation of target proteins including the NF-κB inhibitor proteins IkappaB alpha (NFKBIA), IkappaB beta (NFKBIB) and IkappaB epsilon (NFKBIE), allowing free NF-κB dimers to translocate to the nucleus and activate transcription. BTRC also mediates ubiquitination and subsequent proteasomal processing of the phosphorylated NFKB1/p105 and NFKB2/p100 precursor proteins that respectively participate in the canonical and non-canonical NF-κB signaling pathways [49,50]. If BTRC is a true target of NF-κB, this would suggest that upregulation of BTRC by NF-κB could set up a positive feedback loop to amplify degradation of IkappaB alpha, thereby providing a novel means for magnifying NF-κB signaling. Overall, these results highlight a potential new role for NF-κB in promoting protein ubiquitination by modulating expression of E2 ubiquitin-conjugating enzymes and E3 ubiquitin ligases, thus expanding on its previously documented role in inducing expression of deubiquitinases, proteasome activators and proteasome subunits.

OHMM also revealed new insights into the cross-talk between NF-κB and other signaling pathways. For example, we identified κB sites in several key mediators in the Notch signaling pathway that is involved in cell-cell communications to regulate a broad spectrum of cell-fate determinations. These include the delta-like 1 ligand for Notch receptors DLL1, the Notch2 receptor, transcriptional regulator RBP that acts as a transcriptional repressor in absence of Notch but is a transcriptional activator when associated with activated Notch, and mastermind-like 2 (MAML2) that serves as a transcriptional coactivator for Notch. Prior studies have suggested multiple cross-talk mechanisms between the Notch and NF-κB signaling pathways, most of them suggesting activation of NF-κB downstream of Notch, as well as protein-protein interactions between components of these two pathways (reviewed in [51]). Our finding that key mediators of Notch signaling harbor κB sites supports the notion that NF-κB might in turn modulate Notch signaling to influence cell fate determination during development, immunity and cancer. This would be consistent with recent work showing that NF-κB stimulates the expression of Notch targets HES-5 and Deltex-1 to stimulate marginal zone (MZ) B-cell development [52] and with prior observations from our group that NF-κB can trigger Notch signaling by inducing expression of the Notch ligand Jagged1, although it remains to be determined if its effect on Jagged1 expression is direct [53].

Other genes uncovered by OHMM could be intimately associated with NF-κB's role in transcriptional regulation, and in particular, with its recently uncovered role in gene-specific transcriptional repression [36,54,55]. For example OHMM predicts κB sites for deacetylases HDAC8 and SIRT1, as well as for transcriptional corepressor SIN3A. While NF-κB is known to engage in protein-protein interactions with histone deacetylases and corepressors to modulate gene transcription, OHMM raises the possibility of a new mode of action for NF-κB in this context.

Also highlighted were candidate targets in xenobiotic metabolism, including κB sites in sulfotransferase SULT1C2, aldehyde dehydrogenase 3 family gene ALDH3B2, and transcription factor nuclear factor erythroid derived 2-like 2 (NFE2L2) that regulates the oxidative stress response, in addition to the previously documented NF-κB target glutathione S-transferase (GSTP1; [56]). This points to a possible role for NF-κB in drug metabolism, multidrug resistance and detoxification of poisonous compounds, with possible impact for treatment of infectious diseases, anti-cancer therapy and/or environmental science.

Finally, OHMM pinpointed possible involvement of NF-κB in new pathways, such as the neurotrophin/Trk signaling cascade, in which its role is emerging. In addition to the recently recognized NF-κB target phosphatidylinositol 3-kinase catalytic subunit PIK3C3 [57], OHMM identified κB sites for its regulatory subunit PIK3R2, as well as for the ras-related G-protein RRAS, ribosomal protein S6 kinase 1 RPS6KA1 and transcription factor cyclic AMP-responsive element-binding protein 1 (CREB1). In this regard, CREB3 (better known as LZIP-alpha) was recently described to be controlled by NF-κB [58] as is the critical neurotrophin nerve growth factor NGF that controls neuronal cell fate [59]. These results may warrant further investigation in view of the emerging role of NF-κB in the regulation of neuronal survival and function in the nervous system, its effect on cognition and behavior and its suggested roles in epilepsy, stroke, Alzheimer's, Parkinson's diseases and other neurological disorders (reviewed in [60,61]).

### Verification of some of the predicted NF-κB sites in the human genome

Gel shift experiments were performed on several of the predicted NF-kB sites using two of the NF-κB family members: RelA and c-Rel. Three low occupancy sequences were chosen as negative control and the known *NFKB1A *site was taken as the positive control. We chose the predicted target sites to be tested from some of the top categories that were extracted by pathway analysis. The sites to be tested were from the promoters of following genes: *IKBKB *and *TLR7*, involved in the NF-kB signaling pathway; *DIABLO*, *TANK*, *BCL2L10 *and *BNIP3*, involved in the death receptor signaling and apoptosis categories; *HDAC8*, *SIRT1 *and *SIN3A*, involved in cell cycle G1/S and transcriptional regulation; and *BTRC*, *UBE2D3 *and *UBE2M*, involved in ubiquitination pathway.

**Figure 10:**
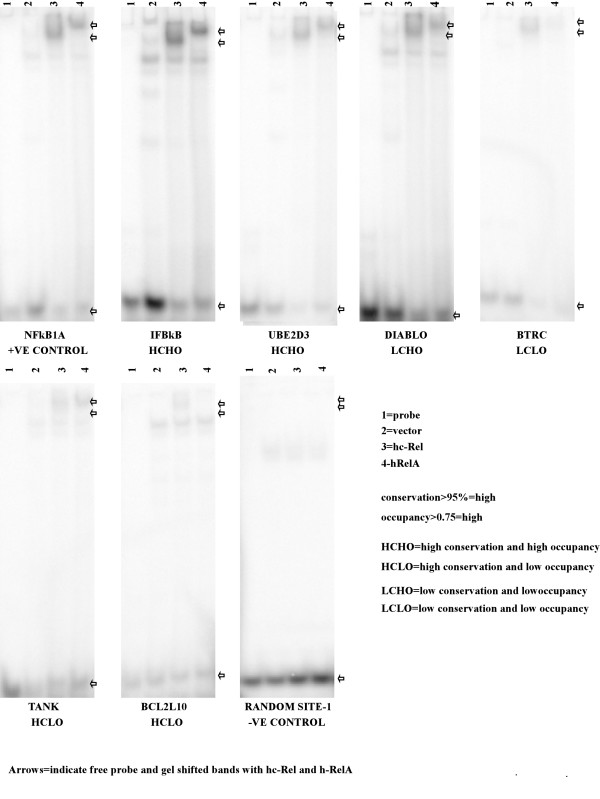
**Gel-shift assays for selected human sites from OHMM predictions**. We show the gel-shift results for representative sites out of the OHMM-predicted locations of high NFκB occupancy in human promoters. NFκBIA site is the positive control. Negative control to the bottom right corresponds to the sequence called site 1 in the result section (AACCACAACCTGCAGCTATTA). Note that lane 3 and lane 4, corresponding to gel shifts with extracts from cells over expressing hc-Rel and hRelaA, respectively, strong shift. Control lane 1 with only the probe (no TF) shows no shift. Lane 2, the other control, represents gel shift with extracts from cells with only the vector. These extract may have some indigenous NFκB from the cell, but the results show very weak shifts compared to the results from lane 3 and 4 coming from the over expression of particular NFκB proteins. The negative control shows that these are results of sequence specific binding.

All of these sites show stronger gel shift compared to the negative controls. To understand which whether predicted occupancy is a good indicator of binding compared to, say, evolutionary sequence conservation, we decided to classify these sites as the following as shown in Figure 10. The conservation score greater than 90% is considered high, between 90% and 80% is considered to be medium and less 80% is considered to be low. Similarly, sites with occupancy greater than 0.75 is considered to be high occupancy and the rest are considered to be low occupancy.

The tested sites, organized according to this classification, are listed below.

High conservation and high occupancy: IFBKB, UBE2D3 and SIN3A

High conservation and low occupancy: TANK-1, TANK-2, BCL2L1 and UBE2M

Medium conservation and high occupancy: SIRT1

Medium conservation and low occupancy: TLR7, HDAC8 and BTRC

Low conservation and high occupancy: BIVIP1 and DIABLO

In general, the high occupancy sites are better binders, the exceptions being SIN3A (weak), HDAC8 (strong) and TLR7 (strong). For HDAC8, the occupancy is only slightly lower than the cut-off threshold. In comparison, most highly conserved low occupancy sites have weak gel shifts, suggesting OHMM occupancy measure is a better predictor of binding affinity than the sequence conservation score.

## Discussion

While the use of an HMM is thus advantageous in identifying sites, we have discovered that an HMM offers tremendous benefits for the special case of identifying self-overlapping motifs. When putative sites overlap, the overall occupancy probability in a position in the DNA sequence depends upon the strength of all sites containing that position. Calculation of the overall occupancy probability over the entire sequence using the gamma variables in the HMM method enables us to compute the occupation probability in presence of overlapping sites. In this regard, our approach has a distinct advantage over the two HMM scoring methods in vogue at the present time, viz. the likelihood method and the Viterbi method. The likelihood of a sequence scored over a long stretch of sequence is greater when more sites are present in that sequence. It is perfect for discovering clusters of sites, as evident from the work on fly regulatory modules. The likelihood score by itself does not indicate the location of sites in a promoter. In contrast, the Viterbi method proclaims the presence of sites at positions where the state path with the highest probability contains the motif state. It, however, fails to consider overlapping sites because only the best site would be present in the optimal state path.

Training OHMM is a non-trivial two-step process. In the first step, we trained both the emission and transition probabilities using short sequences rich in known sites with the aim of accurately estimating the motif profile. In the second step, we kept the emission probabilities constant, and trained the transition probabilities on promoters containing known sites as well as random promoters to accurately estimate the transition probability to the motif *z *reflecting the site density in the promoters of all genes in the human genome. Unlike most HMMs in the literature, we successfully trained the HMM emission probabilities without requiring pre-alignment of training sites.

One interesting feature is that trained *z *is inversely related to the strength of the motif profile. In other words, when the motif profile is kept constant and only *z *is trained, the weaker the motif profile used (i.e. closer to the background), the higher the trained *z*. This is probably the result of the compensating mechanism between the motif profile and *z *discussed above. Interestingly, this competition between *z *and the motif profile also determines the occupancy probability as we saw before.

The goal of the second training step was to estimate the transition probability to the motif *z *reflecting the site density in the promoters of all genes in the human genome. This *z *corresponds to the appropriate threshold when identifying sites in all human promoters. Obviously, *z *trained in the first step was not appropriate due to the high site density in the site-rich training sequences. Instead of training *z *on all human promoters, which is computationally expensive, we train it on a sufficient number of promoters to get a reasonable estimate of *z*. Our procedure of beginning with the human promoters containing known sites as the training set and progressively adding promoters of randomly selected human genes to the training set leads to decreasing *z*, until the training set reached a few thousand promoters and *z *stabilized (Figure 3B). We expect that, for a particular transcription factor, the speed of convergence of this procedure depends upon overall number of target genes of the factor: larger the number of targets/sites, quicker the convergence.

One shortcoming of most site identification methods is that they assume equal probability of the site in a window of arbitrary length around the TSS. The majority of known κB sites are located just upstream of the TSS in gene promoters and the number of known κB sites decreases further upstream. Specifically, of the 36 known κB sites upstream of the TSS, 16 are located within 100 bp and 28 are located within 200 bp of the TSS. Liu *et al*. have also made a similar observation in the promoters of NF-κB-regulated immune genes [10]. To counter the claim that such an observation for binding sites may be due experimental bias, we cite Tabach *et al*. who showed in a wide-scale bioinformatic study that functional binding sites are more likely to be present in the 200 bp region upstream of the TSS than any other upstream region for most human transcription factors and specifically for NF-κB [9]. They defined functional binding sites as those over-represented in functionally related genes (in the same Gene Ontology categories) and conserved in related species. To bolster their conclusion, they showed location dependence of binding sites for the transcription factor Myocardin in a controlled experiment. Xie *et al*. also arrived at a similar conclusion based on binding site conservation [62]. Even though the exact reasons for the occurrence of such a phenomenon are not known at the present time, a better interaction of the transcription factor with the transcription machinery if it is bound close to the TSS and the low density of nucleosomes near the TSS [5] heuristically explain why this phenomenon may occur.

While we agree that a great number experiments need to be conducted to definitively prove the location dependence of functional κB sites, we feel that a site identification method must be able to take it into account. Noticing that site density decreases sharply with the upstream disease from the TSS and that transition probability to the motif *z *is proportional to site density, we modelled *z *using an exponential functional form such that the probability of identifying a site decreases further upstream but never reaches zero (and the transition probability matrix varies accordingly). Even though this approach may fail to identify sites in distal promoters and enhancers, we believe that it allows site search in large upstream regions without identifying too many false positives.

A κB site exerts influence on the occupancy probabilities at the positions surrounding the site in either direction. Because overlapping binding sites are usually present when the scoring window is moved by one position, occupancy probability stays high at all positions in that window. When the window is moved by more positions, the occupancy probability at the new positions dips slightly below the average due to the high motif probability at the site. The occupancy probability returns to the average background value when the scoring window moves by ten positions.

The relationship between the occupancy probabilities of sites located close to each other is quite instructive. When two sites are in tandem without any space between them, occupancy probabilities of both of them are lower due to the small motif-to-motif transition probability. Occupancy probabilities are not very high even when the sites are one position apart because a window shift by one position from a κB site usually contains an overlapping κB site. The sites need to be at least two positions apart so that they do not exert significant influence on each other's occupancy probabilities. Please note that in any case, the overall occupancy in a region containing two nearby sites is quite high.

A strong correlation exists between the occupancy probabilities predicted by OHMM and the *in vitro *binding affinity of the NF-κB family members c-Rel and RelA for oligonucleotides in a gel shift experiment (Figure 8). This validates our physical binding model because the HMM-predicted occupancy probabilities appear to correspond to observed binding affinities. In the process of the study, we found how the padding sequence affects the results. The current practice for performing gel shift experiments in the NF-κB community consists of using particular padding sequences around the 10-mer corresponding to a potential κB site (for example see [63-66]). The commonly used padding sequences, however, can form spurious κB sites due to the self-overlapping nature of κB motif, and hence the experiment may not represent binding of the 10-mer in the native promoter. OHMM offers guidance on the selection of correct padding sequences when designing experiments. A gel shift oligonucleotide should have an A 5' to the κB site and a T 3' to the κB site for minimum interference, and that a 3' C should be avoided at all cost. Ideally, oligonucleotides containing a few bases corresponding to those surrounding the κB site in the promoter of the gene should be used to pad the site. This will capture the effects of all the overlapping putative binding sites in the native promoter and will avoid creation of artefacts based on the nucleotides present in the padding sequence.

The NF-κB family of transcription factors consists of some of the most important proteins for an organism's development and survival. In resting cells, inactive NF-κB dimers reside in the cytoplasm, bound to inhibitors in the IκB family [67]. Many different stimuli can activate latent NF-κB/IκB complexes, including pro-inflammatory cytokines that trigger rapid activation of the classical (canonical) NF-κB signaling cascade via the IKK kinase complex [68]. Consequent phosphorylation and degradation of IκB via the proteasome culminates in nuclear translocation of active NF-κB dimers and their binding to κB sites. This commonly results in transcriptional activation, or occasionally in repression, of genes important for the immune and inflammatory response, cell proliferation, adhesion, angiogenesis and inhibition of apoptosis [36,40,54,55,69-71]. The non-canonical (alternative) NF-κB signaling cascade is activated via the lymphotoxin and BAFF receptors, leading to activation of IKKα, nuclear translocation of p52/RelB complexes and plays a role in lymphoid organogenesis and B-cell function.

Hundreds of NF-κB-regulated genes have been identified (reviewed in [37]). Among them, binding of activated NF-κB to the IκBα promoter can trigger its resynthesis, giving rise to an autoregulatory loop that terminates the activation process [72-74]. Consequently, under normal conditions, Rel/NF-κB activation is tightly regulated and transient. However sustained activation of Rel/NF-κB can promote cell survival, proliferation and/or cell death depending upon the nature and the extent of the stimulus, and is implicated in many pathological conditions including immune system disorders, chronic inflammation and cancer [39,75]. Although several NF-κB target genes have been identified, whether other NF-κB-regulated genes are its direct or indirect targets is not known.

In addition to identifying many of these known NF-κB target genes, OHMM pinpointed several novel candidate NF-κB targets that are yet to be shown as controlled by NF-κB. While further studies will be needed to determine which of the candidate genes identified by OHMM are genuine transcriptional targets of NF-κB, these are likely to yield important new insights into the less well-characterized roles of NF-κB in the induction of apoptotic cell death, its recently uncovered role in transcriptional repression, the regulation of protein ubiquitination and its role in other signaling pathways, to name a few. In addition to identifying multiple genes with key roles in haematological and immune cell development and function, OHMM pinpointed κB sites in genes with roles in drug metabolism, skeletal and muscle system development and function, as well as in disease conditions ranging from immune and inflammatory disorders to infectious diseases, cancer, skeletal muscular disorders and neurological diseases (Table 1; see Additional file 5). These findings are consistent with experimental evidence suggesting a role for NF-κB in immunological and inflammatory diseases, cancer and therapy-resistance, in skeletal myogenesis and cachexia, as well as in cognition, behavior and neurological disorders like epilepsy, stroke, Alzheimer's, Parkinson's diseases, and amyotrophic lateral sclerosis (ALS) (reviewed in [76-79]). OHMM is thus a unique tool to shed new light on the multifaceted biological functions of NF-κB and the mechanisms involved, and to identify critical mediators of dysregulated NF-κB activity in human disorders that may ultimately help to develop new approaches for treatment.

Let us finish the discussion by mentioning possible shortcomings of the method. OHMM requires a complicated two-step training procedure, which might only work practically for factors with many targets. We have not considered insertions or deletions inside the motif, making OHMM well suited for the simultaneous training of emission and transition probabilities. In addition, we have considered the transition probability to the motif *z *in the upstream region to decrease exponentially during the scoring procedure. This density function may not be accurate, especially for enhancers. We will need many more sites to establish a more accurate density function.

## Conclusion

Our method is the first attempt to deal specifically with self-overlapping binding motifs, providing guidance in the selection of padding sequences in gel shift experiments. When the HMM is considered as a biophysical model, it interprets the weight matrix as binding energy, the transition probability to the motif as transcription factor concentration and the gamma variable as occupancy probability. Because it uses occupancy probability as the discriminant function, it learns the associated natural threshold in a principled manner during the training procedure. Another unique feature of OHMM is that transition probabilities change with sequence location to reflect site density. Transcription factor's occupancy probability on a site can be accurately estimated using our method, as seen by the high correlation with experimental binding affinities. High evolutionary conservation scores and enrichment in experimentally regulated genes suggest that κB sites predicted by our method might be functional. Our results may provide important new insights into the function and regulation of NF-κB and uncover possible new biological roles for this important transcription factor family.

## Methods

### Calculation of occupancy probability with overlapping sites using an HMM

The probability that a position in a sequence is occupied by a transcription factor or the occupancy probability at that position can be calculated using either first principles or the standard HMM techniques. We first review these two methods for the case of non-overlapping sites [80]. We then apply these methods to the case of overlapping sites, which is a novel feature of this publication, and show how an HMM provides a simpler solution.

For the purpose of demonstration, let us consider a non-overlapping site and assume that binding is allowed only on one orientation. (A more general case is discussed in Additional file 1.) In calculating occupancy probability using first principles, let's denote the motif state, representing the entire site, as *m *and the background state as *b*. The emission probability of the motif state corresponds to its weight matrix, whereas the transition probability to the motif state from either state is the prior probability of the motif *z*. Let *p*(*b*) be the probability that a long sequence *s *does not contain any motifs (i.e. it is all background), and *P*_*j*_(*m*) be the probability that the sequence has one motif *m *starting at the *j*th position. This latter probability can be written as , where the transition probability to the motif state at any particular position in a sequence is small (*z *≈ 0), ℓ is the length of the motif,  is the probability that the nucleotide *α *at the (*j *+ *i *- 1)th position of the sequence is emitted by the *i*th position of the motif state, and  is the probability that the nucleotide *α *at the (*j *- 1)th position of the sequence is emitted by the background state. Note that this formulation fits into the general Bayesian probabilistic framework such that the product of the *w *terms is the likelihood and the product of the *z *terms is the prior. We can write , where (l - *z*)^ℓ ^≈ 1 and  is the probability that the nucleotide *α *at the (*j *+ *i *- 1)th position of the sequence is emitted by the background state. Also,  such that  is the weight matrix score of the motif starting at the *j*th position of the sequence. Then, the occupancy probability at the *j*th position of the sequence is . Thus, both the motif's weight matrix and prior probability are required to determine occupancy probability.

As described in the Discussion section, the probability of a motif at a particular position in a sequence can be thought of as the occupancy probability of the site at that position. The value of the gamma (*γ*) variable of a state at a particular position in the sequence is the probability of that state at that position. Therefore, , where  and  are the *γ *of the motif and background states at that position, respectively. The gamma variable is an intermediate variable computed during Baum-Welch training. For each state at each position, the gamma variable is the normalized product of the forward and backward variables for that state at that position. When calculating occupancy probability, the gamma variable is also calculated during scoring.

When sites overlap, the occupancy probability at a position needs to combine the effect of binding in all sequence windows containing that position. Moreover, an extra motif type, where emission probabilities are flipped from 5' to 3', takes into account the binding strength of the reverse strand sequence. As described in more detail in Additional file 1, occupancy probability at the *j*th position based on first principles is given by , where *k *corresponds to the first position of each sequence window containing position *j*, *m *is the motif type (including the one corresponding to the site on the other strand), ℓ_*m *_is the length the *m*th motif type, and *z*_*m *_is the transition probability to the *m *th motif type.

To determine the occupancy probability in the case of overlapping sites using an HMM, we divide up each motif type into ℓ states each corresponding to one position in the motif. The emission probabilities of each state are the weights corresponding to that position of the weight matrix. Occupancy probability at a position is then simply , which is similar as in the case of non-overlapping sites. We use this simple formula to calculate occupancy probability.

OHMM consists of 21 states: one background state and a state corresponding to each of the ten positions within the κB motif on the two DNA strands. Because the κB motif is not known to contain insertions or deletions, the transition probabilities between the states corresponding to successive positions within the motif on a strand are fixed to one. The nine transition probabilities available for training are the transition probabilities from (i) the motif states corresponding to the last position in the motif on both strands and (ii) the background state to (i) the motif states corresponding to the first position in the motif on both strands and (ii) the background state. The rest of the transition probabilities are fixed to zero. The emission probabilities of the motif states on the two strands are flipped from 5' to 3' so as to represent identical binding irrespective of the motif strand. Because initial probabilities are a special case of the transition probabilities at one edge of the sequences, we do not mention them separately.

### HMM training and scoring

The transition probabilities were initiated using the transition probability to the motif *z *chosen by us. The motif emission probabilities (motif profile) were initiated using the 97 κB sites generated in unbiased experiments and obtained from TRANSFAC 9.3 [4,81-84]. We will refer to this motif profile as the initial motif profile. The promoters were defined as the regions starting at 800 bp upstream of the TSS (transcription start site) and ending at 100 bp downstream of the TSS. The background state's emission probabilities were assigned from the nucleotide distribution of the promoters corresponding to the reference sequences of all human genes in RefSeq Release 19 [85] associated with human assembly hg18, NCBI Build 36.1 available at the UCSC genome bioinformatics site [86,87]. The background probabilities were also used as pseudocounts when generating the motif profile initially and during subsequent training.

The HMM was trained using the Baum-Welch expectation-maximization algorithm [88] in two steps. In the first step, we used two types of site-rich sequences of different lengths as well as various initial *z*'s to train all HMM parameters and determine the emission probabilities to be used in the second step. The following two types of site-rich sequences were used. (1) The "TSS-n promoters" consist of n nucleotides upstream of the TSS of the 42 human genes known to contain a κB site [4,89,90]. (2) The "Surround-n promoters" consist of 34 promoters containing the 36 known κB sites whose exact genomic locations were identified (two promoters each contained two closely located known κB sites) and the surrounding regions. Each surround-n promoter is n nucleotides long. The HMMs trained on these promoters were called "TSS-n HMMs" and "surround-n HMMs," respectively. After each training iteration, the emission probabilities of the motif states of the corresponding motif positions on both strands were averaged to ensure that the learned motif profiles on both strands were exactly flipped 5' to 3'. After training, the sum of the transition probabilities from the background state to the motif states corresponding to the first motif position on both strands was estimated as *z *(the transition probabilities to the motif states on the two strands are nearly identical).

In the second training step, only the transition probabilities were trained. The various training sets consisted of human promoters containing the known κB sites and a different number of randomly selected human promoters added to them. Training in the upstream 800 bp and downstream 100 bp regions with respect to the TSS was done separately.

To identify binding sites, promoter regions 800 bp upstream of the TSS were scored using the HMM *γ *variable and location-dependent transition probabilities. For this, transition probability to the motif *z *was modeled using an exponential functional form such that a region close to the TSS had a higher site density than a region further upstream. The mean of this exponential functional form was estimated as follows. *z *can be written as  at *x *positions upstream of the TSS, where *θ *is the mean distance of κB sites upstream of the TSS (in number of nucleotide positions) and *z*_0 _is the scale factor. Based on the position of known upstream κB sites, the maximum likelihood estimate of the mean for the exponential form was 170, and the estimate of the mean using the median was quite close at . The maximum likelihood estimate was used in further analysis. To determine the scale factor *z*_0_, we noted that the site density per promoter is 800* _*z *_if *z *has a uniform functional form and  if *z *has an exponential functional form. Equating these two expressions for uniform *z *= 0.00017 obtained from training and *θ *= 170 results in z_0 _= 0.137.

Location-dependent transition probabilities based on the above calculations were used to compute occupancy probability (*γ *variable) and identify sites in the upstream promoter regions. The value of *z *was calculated at each upstream position. Accordingly, a different transition probability matrix was generated at each upstream position as follows. We assigned the transition probability from (i) the background and (ii) the motif states corresponding to the last motif position on either strand to the background state as 1 - *z*, and the transition probability from (i) the background and (ii) the motif states corresponding to the last motif position on either strand to the motif states corresponding to the motif first position on either strand as *z*/2. The forward (*α*) and backward (*β*) variables were calculated using the standard HMM recursion relations incorporating the position-specific transition probabilities (for example, the probability of observing the partial sequence *O*_1_...*O*_*t *_until position *t *and being in state *j *at position *t *is given by the recursion relation , where the transition probability *α*_*ij *_from state *i *to state *j *varies with position *t*, *b*_*j*_(*O*_*t*_), is the emission probability of state *j *that generates the nucleotide at position *t *and *n *is the number of states). The probability of state *i *at position *t *is the gamma variable . The 100 bp regions downstream of the TSS were scored using the HMM *γ *variable and location-independent transition probabilities with *z *of 0.00012 (as obtained from training for these regions).

We compared the performance of OHMM to that of a weight matrix (WM) as follows. WM scoring was performed using the initial motif profile. All overlapping windows on both strands were considered and the highest WM score was recorded. Positive examples consist of the 36 known human κB sites present in upstream 800 bp regions (in their native promoters). Negative examples consist of all 10-mers in the upstream 800 bp regions in 100 randomly selected human genes that have no association with inflammation or cancer. Leave-one-out cross-validation was performed, where each site was scored using an HMM trained on the surround-50 promoters of the other 35 known κB sites. The lists of HMM and WM scores of the negative examples were compressed by taking the maxima of the consecutive scores above a threshold (0.03 for HMM, 4 for WM) to ensure that overlapping binding sites were represented by the score of the strongest site.

### Conservation, expression and functional annotation

To calculate the conservation score of a site, its multiple alignment was retrieved from UCSC. Only mammalian sequences with at least five nucleotides present in the alignment were included. Consensus nucleotides were determined at all positions in the alignment where the human sequence did not contain a gap, and the number of sequences containing the consensus nucleotide was counted for each position. The conservation score was calculated as the ratio of the sum of these counts at all positions to the product of the number of sequences in the alignment and the number of nucleotides in the site (generally 11 or 12 for overlapping κB sites, 10 for non-overlapping κB sites), multiplied by 100. The perfect score, when all aligned sequences are identical, is 100. Kernel-smoothing density estimates of the conservation scores of sets of κB sites were calculated using default MATLAB parameters.

We used the chicken microarray experiment described in [36] to determine if chicken genes regulated by over-expressed NF-κB proteins were enriched with κB sites predicted by OHMM. Human orthologs of the regulated chicken genes were obtained using Ensembl [91].

For comparison with gel shift binding affinities, occupancy probabilities need to be calculated based on an accurate transition probability to the motif (*z*), which corresponds to the protein concentration as described in the Discussion section. However, protein concentration in a gel is higher than in the cellular context, and is difficult to determine. We therefore estimated *z *as follows: (1) calculate the occupancy probabilities of all the sequences in the gel shift experiment using various *z*'s, (2) compute the sum of KL divergences of the occupancy probabilities of all the sequences with their binding affinities in the gel shift experiment, and (3) estimate *z *as the one corresponding to the minimum sum. The rationale behind this procedure is that the occupancy probabilities resulting from the correct *z *should be in the same ballpark range as gel shift binding affinities. KL divergence can be used as a measure to determine if they are indeed in the same ballpark range. The estimated *z *is 0.001 for both RelA and c-Rel.

To determine the cellular pathways, biological functions and diseases in which the predicted NF-κB targets were over-represented, genes containing κB sites with predicted occupancy probability greater than 0.5 were analyzed through the use of Ingenuity Pathways Analysis (Ingenuity^® ^Systems, ) and DAVID [92-94].

The computational analysis was performed using Perl and MATLAB (version R2006b, The MathWorks, Inc.). The κB motif logos were generated using MATLAB [95].

### Gel shift experiments for chicken sequences

Gel shift assays were performed as described [96] with protein lysates (15 μg) from human 293T cells transfected with either c-Rel or RelA. Radiolabeled double-stranded oligonucleotide probes contained either a palindromic κB DNA site [63], or the predicted NF-κB sites from chicken *blnk *site 1 (GTGGCTTCCC), or *blnk *site 2 (CGGGATCCCC); *pdcd4 *(CGGGCGTCCC); *itm2b *(GGGAGATTCC), *pp1e *(GGGGATGTCC), *bcap *(TGGGATCCCC), *igλ *(GGGGCATCCC), or *mip-1β *(GGGGTTTCCC) with the 5' padding sequence GATCTGAATTCGT and the 3' padding sequence CACCTCTCCTTA.

### Gel shift assays for human sequences

Gel shift assays were performed as in [96] with the following modifications. Protein lysates used were the same as described for the chicken sites except 4 μg of protein was used. Radiolabeled double-stranded oligonucleotide probes were prepared by annealing a shorter 9 bp oligo, which is complimentary to 3'end of the 21 bp longer oligo and by incorporating α^32^P dCTP and α^32^P dATP and cold isotopes during a fill in reaction using Klenow Polymerase. The probes were purified using Amersham Microspin G-25 Columns (Cat# 27-5325-01) according to the manufacturer's protocols. The radio-labeled double stranded oligonucleotide probes, with the native padding sequences on either side (taken from the human genome), consist of the *NFKB1A *site as the positive control (TGGCT**TGGAAATTCCC**CGAGC); the predicted NF-kB sites from human *IFBKB *(GGCGC**GGGAAATTCCA**CCGAG); *UBE2D3 *(AGTCT**GGGGAATTCCA**TTTCC);*SIN3A *(TGGGC**GGGATTTCCC**GGGTA); *TANK-1 *(CTCAG**TGGAAGTTCCC**ACTTC); *TANK-2 *(AAGTT**GGGGGATTTCTC**AGTGG); *BCL2L10 *(GCAGG**TGGGATTCCCA**TCAAA);*UBE2M *(TGAAC**GGAAATGCCC**GAGTC); *BNIP1 *(GTCAG**GGAAAGTCCC**AACTC); *DIABLO *(CACCA**GGAAATTCCC**TTCAA); *SIRT1 *(AGACG**TGGAAATTCCC**AGGGC); *TLR7 *(TAGTT**GGAAACTCCA**GGGCT); *HDAC8 *(GGTCT**GGGAAGTCCC**ATCCA); *BTRC *(CCTGG**GGGAAGTTCC**AGAAC) and 3 negative control sites, site 1 (AACCACAACCTGCAGCTATTA); site 2 (AAAATGTGGTAGATAATGGTG); and site 3 (TAAGTAAACATGATATTAGGA).

The binding reaction was performed exactly as described for chicken sites and the Products were resolved on an 8% gel. The gels were run in 0.25× TBE buffer for 3 hrs at 150 volts. The gels were wrapped in thin plastic sheet protectors and exposed to a Molecular Dynamics Phosphoimager.

## Authors' contributions

AD and AMS developed the HMM. AD performed the computational work. NG carried out the chicken gel shift assay under CG's supervision. VN performed the human gel shift assay. CG evaluated the biological significance of the results. All authors read and approved the final manuscript.

## Supplementary Material

Additional file 1 **Derivation of occupancy probability of overlapping sites**. The file contains a derivation of occupancy probability in the case of overlapping sites using first principles. It also shows that even though a conventional weight matrix and an HMM are closely related, an HMM is more appropriate to determine occupancy probability when overlapping sites exist. Click here for file

Additional file 2 **HMM emission probabilities**. The file contains the emission probabilities of an untrained HMM as well as of the trained surround-n HMMs. Unused emission probabilities based upon known kB sites present in the surround-n promoters are also provided for reference. Click here for file

Additional file 3 **Enrichment of regulated genes with predicted sites**. The file contains the number and percentage of chicken genes and their human orthologs with at least one predicted site above various thresholds. Data is shown for all genes as well as for genes regulated in at least 2 or 4 comparisons in the microarray experiment. For genes regulated in at least 4 comparisons, the number of predicted sites as well as the number of sites expected based upon all genes in the genome at various thresholds are also shown. Click here for file

Additional file 4 **Predicted sites in human genes**. The file contains sites in all human genes that are predicted by OHMM to have occupancy probability greater than 0.5. The file also contains relevant annotation. Click here for file

Additional file 5 **Enriched biological categories**. The file contains cellular pathways, biological functions, diseases and toxicology-related lists enriched with HMM-predicted NF-κB targets. Click here for file
