# Supplementary material for: OHMM: a Hidden Markov Model accurately predicting the occupancy of a transcription factor with a self-overlapping binding motif
Source: BMC Bioinformatics. 2009 Jul 7;10:208. doi: 10.1186/1471-2105-10-208 (PMC2718928; doi:10.1186/1471-2105-10-208)
Supplement: Additional file 1 — Derivation of occupancy probability of overlapping sites. The file contains a derivation of occupancy probability in the case of overlapping sites using first principles. It also shows that even though a conventional weight matrix and an HMM are closely related, an HMM is more appropriate to determine occupancy probability when overlapping sites exist. [file 1471-2105-10-208-S1.doc]

### Additional file 1 – Derivation of occupancy probability of overlapping sites

In this text, we will calculate the occupancy probability of sites at any position in a sequence as well as over the entire sequence using first principles. We will consider the cases of one site, non-overlapping sites of the same type, exactly overlapping sites of multiple types and finally the general case of overlapping sites of multiple types. We will also show that even though a conventional weight matrix and an HMM are closely related, an HMM is more appropriate to determine occupancy probability when self-overlapping sites exist.

We will use the following symbols in the derivation below: *b* = background, *m* = motif where motif is a representation of a binding site, $\alpha$ = nucleotide, = length of a motif, *i* = position in a motif, *z* = transition probability to the motif, *s* = sequence, *L* = length of the sequence, *j* = position in the sequence, $w_{\alpha}^{bj}$ is the probability that nucleotide$\alpha$ at the *j*’th position of the sequence is emitted by the background, $w_{i\alpha}^{mj}$ and $w_{i\alpha}^{bj}$ are the probabilities that nucleotide$\alpha$ at the $\left(j+i-1\right)$’th position of the sequence is emitted by the *i*’th position of the motif or by the background respectively, $p_{j}(s)$ is the occupancy probability of transcription factors at position *j* of a sequence and $p\left(s\right)$ is the occupancy of transcription factors over the entire sequence. Because most of the promoter sequence is background, transition probability to the motif $z\approx0$ and hence .

The weight matrix score corresponding to the motif starting at the *j*’th position of the sequence is defined as

(A1)

The motif’s strength compared to the background at that position is (A2)

## One Site

When a sequence *s* is scored using an HMM, the likelihood of the sequence is the sum of all configurations, i.e. combinations of background and motif states at all positions of the sequence. The configuration with the background state at all positions has the probability

$p(b)=\ldots\left(1-z\right).w_{\alpha}^{bj}.\left(1-z\right).w_{\alpha}^{b\left(j+1\right)}\ldots=\left(1-z\right)^{L}.\prod_{j=1}^{L} w_{\alpha}^{bj}$… (A3)

The configuration with motif *m* at the *j*’th position has the probability

and can be expressed in terms of the probability of all background as follows: (A4)

Thus, the two factors$z$and $E_{j}=e^{W_{j}}$, one the transition probability to the motif and the other a measure of distinctness of the emission probabilities of the motif from that of the background, determine occupancy probability. Occupancy probability at position *j* in terms of the transition probability to the motif and the weight matrix score is given by

… (A5)

As long as we know *z*, we can calculate the occupancy probability at a sequence position using the weight matrix, and hence the weight matrix threshold for classifying sequences into sites can be easily determined from the occupancy probability threshold. For example, occupancy probability threshold of 0.5 (corresponding to $p_{j}(m)=p(b)$ and $z.e^{W_{j}}=1$) results in the weight matrix threshold of $W=-\ln z$.

We can also calculate the occupancy probability using an HMM. The HMM gamma variable corresponds to the probability that position *j* of the sequence is in certain state. For example, $\gamma_{j}^{m}$ and $\gamma_{j}^{b}$ correspond to the motif and background states respectively, such that $\gamma_{j}^{m}+\gamma_{j}^{b}=1$ if only these two states are considered. Hence, gamma of the motif state at a sequence position is the occupancy probability at that position ($p_{j}(s)=\gamma_{j}^{m}=1-\gamma_{j}^{b}$).

## Non-overlapping sites of the same type

A configuration containing two non-overlapping sites of the same type at positions $j_{1}$ and $j_{2}$ has the probability

For the sake of explanation, considering only non-overlapping sites is equivalent to considering a motif at only one position emitting bases with each position having one of the two states *m* or *b*.

Let’s calculate the overall likelihood of the sequence to understand the relationships between the different terms. The likelihood is the sum of the probabilities of all configurations: $e^{L}=p(b_{1}b_{2}\ldots)+p(b_{1}m_{2}\ldots)+p(m_{1}b_{2}\ldots)+\ldots$

When the transition probability to the background or the motif is independent of the previous state,

$$e^{L}=p(b_{1}).p(b_{2}\ldots)+p(b_{1}).p(m_{2}\ldots)+p(m_{1}).p(b_{2}\ldots)+\ldots$$

$$e^{L}=\left(p(b_{1})+p(m_{1})\right).\left(p(b_{2}\ldots)+p(m_{2}\ldots)\right)$$

$$e^{L}=\prod_{j=1}^{L} \left(p(b_{j})+p(m_{j})\right)$$

… (A6)

… From equation (A3)

$e^{L}\approx\left(1-z\right)^{L}.\prod_{j=1}^{L} w_{\alpha}^{bj}.\prod_{j=1}^{L} \left(1+z.E_{j}\right)$… Because

$e^{L}\approx\left(1-z\right)^{L}.\prod_{j=1}^{L} w_{\alpha}^{bj}.\left(1+\sum_{j}^{L} z.E_{j}+\sum_{j}^{L} \sum_{k\neq j}^{L} z.E_{j}.z.E_{k}+\ldots\right)$… (A7)

In equation (A7), the first term corresponds to the configuration with only background, the second term corresponds to all configurations with one site, the third term corresponds to all configurations with two non-overlapping sites, etc. Note that the summation terms in equation (A7) do not take overlapping sites into account and hence the above equations are inaccurate for overlapping sites.

We see from equation (A6) that the likelihood is dominated by high *E_j_*’s. If there is only one strong weight matrix score ($z.E_{j}=z.e^{W_{j}}>>1$), the likelihood is in the order of magnitude of its exponent. If there are multiple strong weight matrix scores, the likelihood is in the order of magnitude of the product of their exponents. However, if there are many moderate weight matrix scores ($z.E_{j}\approx1$), the likelihood will also increase slightly. Most weight matrix scores are very low ($z.E_{j}<<1$) and thus do not contribute to the likelihood.

To determine the occupancy over the entire sequence, let’s calculate the maximum likelihood estimate (MLE) of *z* by taking the derivative of log likelihood.

$$\begin{matrix} L\approx L.\ln\left(1-z\right)+const+\sum_{j=1}^{L} \ln\left(1+z.E_{j}\right) \\ \frac{\partial L}{\partial z}=-\frac{L}{1-z}+\sum_{j=1}^{L} \frac{E_{j}}{1+z.E_{j}} \\ L=\sum_{j=1}^{L} \frac{E_{j}}{1+z.E_{j}} \end{matrix}$$

Therefore, occupancy over the entire sequence, i.e. the product of the sequence’s length and the transition probability to the motif is

$p\left(s\right)=L.z=\sum_{j=1}^{L} \frac{z.E_{j}}{1+z.E_{j}}$ … (A8)

As with the case of calculating the occupancy probability at a position, the knowledge of z allows us to calculate the occupancy over the entire sequence with the help of a weight matrix. In the HMM context, this is simply the sum of occupancies at all positions, i.e. the sum of gammas of the first position of the motif at all positions ($p\left(s\right)=\sum_{j=1}^{L} \gamma_{j}^{m_{1}}$).

## Exactly overlapping sites of multiple types

When different types of sites are present such that they overlap exactly, for example when we consider the same site on both strands as in the case of NF-κB, the occupancy probability of any type of site at a position is

$p_{j}(s)=\frac{p(m1)+p(m2)}{p(b)+p(m1)+p(m2)}\approx\frac{z_{1}.E_{1j}+z_{2}.E_{2j}}{1+z_{1}.E_{1j}+z_{2}.E_{2j}}=\frac{\sum_{m} z_{m}.E_{mj}}{1+\sum_{m} z_{m}.E_{mj}}$… (A9)

where *m* indicates the motif type, representing the type of the site, and $E_{mj}$ is the exponent of the weight matrix of motif type *m* starting at sequence position *j*. In this case, calculation of occupancy probability using weight matrices requires knowledge of multiple *z*’s. In the HMM context, however, the occupancy probability is simply the sum of gammas of all motif states, or alternatively$p_{j}(s)=1-\gamma_{j}^{b}$, where $\gamma_{j}^{b}$ is the gamma of the background at that position.

The likelihood of the entire sequence is then

$$e^{L}=\prod_{j=1}^{L} \left(p(b_{j})+p(m1_{j})+p(m2_{j})\right)$$

$$e^{L}\approx p(b).\prod_{j=1}^{L} \left(1+z_{1}.E_{1j}+z_{2}.E_{2j}\right)=p(b).\prod_{j=1}^{L} \left(1+\sum_{m} z_{m}.E_{mj}\right)$$

The occupancy probability of any type of site over the entire sequence is $p\left(s\right)=\sum_{j=1}^{L} \frac{\sum_{m} z_{m}.E_{mj}}{1+\sum_{m} z_{m}.E_{mj}}$. Its calculation using weight matrices is tedious. However, it can be easily calculated using HMMs as $p\left(s\right)=\sum_{j=1}^{L} \sum_{m} \gamma_{j}^{m_{1}}$, where $\gamma_{j}^{m_{1}}$ is the gamma of the first position of the *m*’th type of motif at the *j*’th position of the sequence.

## Overlapping sites of multiple types

Finally, we consider the most general case: overlapping sites of multiple types. The probabilities of configurations of two self-overlapping sites are shown below:

Hence, to calculate the occupancy probability at a position in the case of self-overlapping sites, we need to consider all windows containing the position. Similar to equation (A9), occupancy probability at a sequence position in the case of self-overlapping sites is:

… (A10)

where *k* is the first position of each sequence window containing sequence position *j*. Extending equation (A10), occupancy probability at a position in the case of overlapping sites of multiple types is:

… (A11)

where *m* is the motif type (including the motif on the other strand). Because this occupancy probability depends upon multiple sequence windows, its calculation using weight matrices is not straightforward even when we know the *z*’s. On the other hand, the HMM gamma variable automatically takes the overlaps into account, and hence the occupancy probability using an HMM is simply

$p_{j}(s)=1-\gamma_{j}^{b}$ … (A12)

where $\gamma_{j}^{b}$ is the gamma of the background at that position. This is the same formula as for non-overlapping and exactly overlapping sites. The occupancy probability by a particular motif type is simply its gamma value ($\gamma_{j}^{m}$).

Calculation of the occupancy over an entire sequence in the above case is quite difficult using weight matrices and quite easy using an HMM. A single configuration cannot contain overlapping sites. Therefore, no simple formula for the overall likelihood of the sequence exists (for example, the second term in equation (A7) is invalid). Hence, the knowledge of the *z* and the weight matrix does not allow us to calculate the overall occupancy of the sequence quickly. However, exactly as for non-overlapping and exactly overlapping sites, we can easily calculate the overall occupancy using an HMM as

$p\left(s\right)=\sum_{j=1}^{L} \sum_{m} \gamma_{j}^{m_{1}}$ … (A13)

where $\gamma_{j}^{m_{1}}$ is the gamma of the first position of the *m*’th type of motif at the *j*’th position of the sequence.

In the absence of overlapping sites, the probability of occupancy calculated using the weight matrix score and the *z* , the HMM gamma of the first state of the motif and the HMM gamma of the entire motif are identical. However, in the case of overlapping sites, the HMM gamma of the entire motif is higher than the probability of occupancy calculated using the weight matrix score and the *z* because the latter fails to consider the overlapping sites. It is in turn higher than the HMM gamma of the first state of the motif, which has a low value due to the presence of a site in an overlapping window.
